# Age-associated nicotinamide adenine dinucleotide decline drives CAR-T cell failure

**DOI:** 10.1038/s43018-025-00982-7

**Published:** 2025-05-20

**Authors:** Helen Carrasco Hope, Jana de Sostoa, Pierpaolo Ginefra, Massimo Andreatta, Yi-Hsuan Chiang, Catherine Ronet, Christine Pich-Bavastro, Jesús Corria Osorio, François Kuonen, Johan Auwerx, Patrizia D’Amelio, Ping-Chih Ho, Santiago J. Carmona, George Coukos, Denis Migliorini, Nicola Vannini

**Affiliations:** 1 Department of Oncology, University of Lausanne, Lausanne, Switzerland; 2 Ludwig Institute for Cancer Research, University of Lausanne, Epalinges, Switzerland; 3 Department of Oncology, Geneva University Hospitals (HUG), Geneva, Switzerland; 4 Agora Cancer Research Center, Lausanne, Switzerland; 5 Swiss Cancer Center Léman (SCCL), Lausanne and Geneva, Geneva, Switzerland; 6 Center for Translational Research in Onco-Hematology, University of Geneva, Geneva, Switzerland; 7 Department of Dermatology and Venereology, Lausanne University Hospital (CHUV) and University of Lausanne, Lausanne, Switzerland; 8 Laboratory of Integrative and Systems Physiology, Institute of Bioengineering, Ecole Polytechnique Fédérale de Lausanne (EPFL), Lausanne, Switzerland; 9 Service of Geriatric Medicine and Geriatric Rehabilitation, Department of Internal Medicine, University of Lausanne Hospital Centre (CHUV), Lausanne, Switzerland

**Keywords:** Cancer immunotherapy, Metabolism, Cancer

## Abstract

Chimeric antigen receptor (CAR) T cell therapy is one of the most promising cancer treatments. However, different hurdles are limiting its application and efficacy. In this context, how aging influences CAR-T cell outcomes is largely unknown. Here we show that CAR-T cells generated from aged female mice present a mitochondrial dysfunction derived from nicotinamide adenine dinucleotide (NAD) depletion that leads to poor stem-like properties and limited functionality in vivo. Moreover, human data analysis revealed that both age and NAD metabolism determine the responsiveness to CAR-T cell therapy. Targeting NAD pathways, we were able to recover the mitochondrial fitness and functionality of CAR-T cells derived from older adults. Altogether, our study demonstrates that aging is a limiting factor to successful CAR-T cell responses. Repairing metabolic and functional obstacles derived from age, such as NAD decline, is a promising strategy to improve current and future CAR-T cell therapies.

## Abstract (web summary)

Vannini and colleagues report that an age-associated decline in nicotinamide adenine dinucleotide levels is associated with mitochondrial dysfunction and reduced antitumor efficacy of chimeric antigen receptor T cells derived from older adults.

## Main

Immunotherapeutic strategies based on adoptive cell transfer (ACT) of chimeric antigen receptor (CAR) T cells are currently among the most promising approaches to treat cancer. Amongst the features that determine successful responses to CAR-T cell therapy, the long-term maintenance of a T cell pool with stem-like properties is fundamental^1,2^. As a result, the CAR-T cell field is evolving toward finding strategies that favor the quantity and quality of stem-like T cells in CAR-T infusion products^1–3^. Importantly, this stem-like population engages a particular metabolic program that relies mostly on mitochondrial activity^4–8^. Indeed, recent studies demonstrated that CAR-T cell products with decreased mitochondrial fitness are associated with poor responses^9,10^ and metabolic interventions boosting mitochondrial metabolism are able to improve the efficacy of CAR-T cell therapy in preclinical models^11–15^.

A key metabolite that ensures mitochondrial health is nicotinamide adenine dinucleotide (NAD). NAD is a well-known cofactor that has a pivotal role in redox balance and energy metabolism by fueling oxidative phosphorylation. Moreover, it serves as a substrate for several enzymes, such as sirtuin deacetylases (SIRTs), that can regulate the expression of peroxisome proliferator-activated receptor-γ coactivator 1α, a transcription cofactor involved in mitochondrial biogenesis^16–18^. NAD metabolism critically regulates T cell fate and function^19–21^. Thus, alterations in NAD homeostasis have been linked to impaired T cell responses^20,22^, while restoration of mitochondrial dysfunction through NAD-boosting strategies has been shown to prevent exhaustion of tumor-infiltrating lymphocytes (TILs)^23^.

Aging is the first risk factor associated with cancer. Consequently, the majority (~75%) of persons with cancer and persons eligible for cancer immunotherapy are >65 years old. Importantly, in the context of CAR-T cell therapy, the highest response is observed in B cell acute lymphoblastic leukemia (B-ALL) when the median diagnostic age is <20 years old, while the responses decline with increasing age^24^. However, whether aging is an important limiting factor for CAR-T cell efficacy and its underlying mechanisms is still unknown. Several investigations have reported that aging leads to deficient immune and metabolic functions that result in altered antitumor responses^25^. Interestingly, mitochondrial dysfunction is a hallmark of aging^26^ and NAD decline has been described across several tissues including white adipose tissue (WAT), muscle and liver^27–29^. In our study, we demonstrate that age is a limiting factor for effective CAR-T cell responses. We show that aging drives mitochondrial dysfunction in T cells, which impairs their stem-like properties and antitumor capacities when transduced with a tumor-antigen-directed CAR. We then determine the decline in NAD cellular levels as a major factor responsible for this process and the restauration of NAD homeostasis as a strategy to rejuvenate old CAR-T cells.

## CAR-T cells from aged mice have limited stem-like properties

**Fig. 1:**
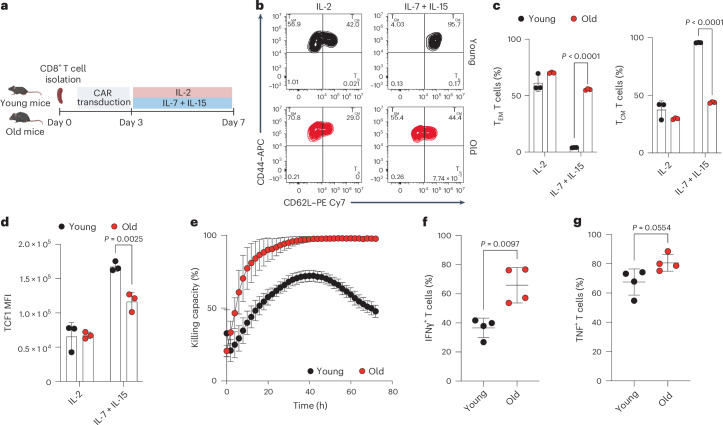
CAR-T cells generated from aged mice are unable to preserve stem-like properties. **a**, HER2-directed CAR-T cells were generated from CD8^+^ T cells isolated from spleens of young (8 weeks old) and old (>80 weeks old) mice. On day 3, T cells were further expanded in the presence of IL-2 or IL-7 and IL-15. **b**, Representative CD44 and CD62L dot plots of young and old CAR-T cells on day 7 upon expansion with IL-2 or IL-7 and IL-15. **c**, Proportion of CAR-T cells with T_EM_ (CD44^+^CD62L^−^) and T_CM_ (CD44^+^CD62L^+^) phenotype (*n* = 3 biologically independent samples). **d**, Levels of TCF1 within the T_CM_ population of young and old CAR-T cells on day 7 (*n* = 3 biologically independent samples). **e**, Killing capacity of young and old CAR-T cells expanded with IL-7 and IL-15 upon coculture with B16-HER2 cells at a 2:1 effector-to-target ratio. The graph is representative of three independent experiments; error bars represent technical replicates. **f**, Proportion of IFNγ^+^ CAR-T cells expanded with IL-7 and IL-15 upon coculture with B16-HER2 cells (*n* = 4 biologically independent samples). **g**, Proportion of TNF^+^ CAR-T cells expanded with IL-7 and IL-15 upon coculture with B16-HER2 cells (*n* = 4 biologically independent samples). Data are presented as the mean values ± s.e.m. Statistical analysis was performed using a two-way analysis of variance (ANOVA) with Tukey’s multiple-comparison test (**c**,**d**) or unpaired *t*-test (**f**,**g**), as appropriate. Panel **a** created with BioRender. Source data

**Extended Data Fig. 1:**
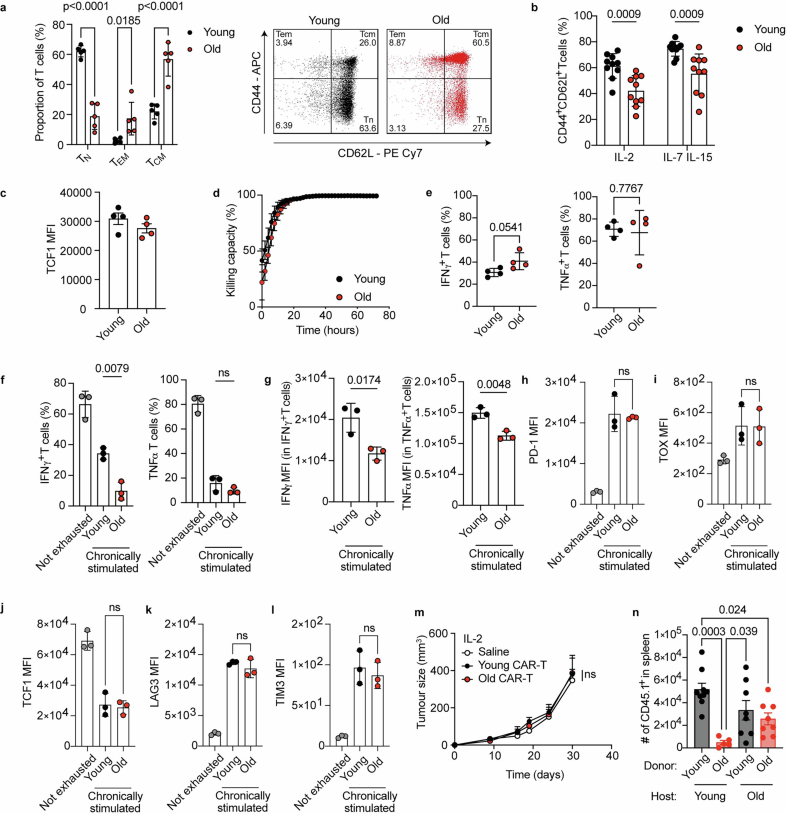
Aged CAR-T cells are unable to maintain stem-like properties. (**a**) Proportion of CD8^+^ T_n_ (CD44^−^CD62L^+^), T_cm_ (CD44^+^CD62L^+^) and T_em_ (CD44^+^CD62L^−^) cells in the spleens of young and old mice. On the right, representative CD44/CD62L dot plot (n = 5 biologically independent samples). (**b**) Proportion of T_cm_ cells (CD44^+^CD62L^+^) upon activation of young and old naïve CD8^+^ T cells and expansion under effector-like (IL-2) or memory-like (IL-7 IL-15) conditions (n = 10 biologically independent samples). (**c**) Mean fluorescence intensity (MFI) of TCF-1 within young and old T_cm_ populations expanded from naïve CD8^+^ T cells with IL-7 and IL-15 (n = 4 biologically independent samples). (**d**) Killing capacity against B16-HER2 tumor cells of young and old CAR-T cells expanded with IL-2 (n = 3 biologically independent samples). (**e**) Proportion of IFNγ^+^ and TNFα^+^ young and old CAR-T cells expanded with IL-2 upon rechallenge with B16-HER2 tumor cells (n = 4 biologically independent samples). (**f**) Proportion of IFNγ^+^ and TNFα^+^ young and old exhausted CAR-T cells generated upon chronic stimulation *in vitro* (n = 3 biologically independent samples). (**g**) MFI of IFNγ and TNFα within IFNγ^+^ and TNFα^+^ populations, respectively, of young and old exhausted CAR-T cells generated upon chronic stimulation *in vitro* (n = 3 biologically independent samples). (**h-l**) Levels of the exhaustion markers PD-1, TOX, TCF-1, LAG3 and TIM3 in young and old exhausted CAR-T cells (n = 3 biologically independent samples). (**m**) Growth of B16-HER2 tumors after ACT of young and old CAR-T cells expanded with IL-2 (n = 6 mice per group). (**n**) CAR-T cells (CD45.1^+^) generated from young or old mice were adoptively transferred into young or old recipient mice bearing B16-HER2 tumors. Number of CD45.1^+^ CD8^+^ T cells were analyzed in the spleens 20 days post-ACT (n = 9 Y > Y, n = 5 O > Y, n = 8 Y > O, n = 8 O > O). Data are represented as mean values +/- SEM. Statistical analysis was performed using two-way ANOVA (A-C), unpaired *t*-test (E, G and M) or one-way ANOVA with multiple comparisons (F, H-I and N), as appropiate. ns = p > 0.05. Source data

To decipher how aging affects the composition of CAR-T cell infusion products, we transduced CD8^+^ T cells derived from young (8 weeks old) and old (>80 weeks old) mice with a Thy1.1^+^ CAR construct targeting the human oncogene human epidermal growth factor receptor 2 (HER2, also known as ERBB2) (refs. ^13,14,30^). CAR-T cells were then expanded under effector-like (T_EM_) or memory-like (T_CM_) polarizing conditions (involving the use of interleukin 2 (IL-2) or IL-7 and IL-15, respectively) (Fig. 1a). Young and old CAR-T cells displayed comparable differentiation capacity when cultured in the presence of IL-2 (Fig. 1b,c). However, old CAR-T cells were unable to properly acquire a memory-like phenotype when cultured with IL-7 and IL-15, as shown by both the lower proportion of T_CM_ cells (Fig. 1b,c) and the decreased levels of the stemness marker T cell factor 1 (TCF1) when compared to younger counterparts (Fig. 1d)^31–33^. To prove that these differences stem not only from the accumulation of experienced (CD44^+^) T cells with age (Extended Data Fig. 1a) but also from cell-intrinsic defects, we isolated CD8^+^ naive T cells (CD44^−^CD62L^+^) from young and old mice and tested their differentiation capability in vitro. Following initial activation, we expanded under T_EM_ or T_CM_ polarizing conditions and we observed that naive T cells derived from aged mice did not differentiate efficiently toward a T_CM_ phenotype (Extended Data Fig. 1b). Accordingly, the T_CM_ population had lower TCF1 expression (Extended Data Fig. 1c). These results suggest that the defective memory-like phenotype identified in aged CAR-T cell infusion products is not only because of differences in the initial population heterogeneity but also because of cell-intrinsic defects of CD8^+^ T cells.

To further assess their functionality, we rechallenged young and old CAR-T cells against HER2-overexpressing B16 melanoma cells in vitro. Aged CAR-T cells exhibited a higher killing capacity than young CAR-T cells (Fig. 1e) and an increased capacity to produce interferon-γ (IFNγ) and tumor necrosis factor (TNF) (Fig. 1f,g) when compared to young CAR-T cells, thus reflecting the acquisition of a T_EM_ phenotype. Of note, no differences were observed between young and old CAR-T cells when cultured in the presence of IL-2 (Extended Data Fig. 1d,e). Despite providing enhanced cytotoxicity, T_EM_ cells exhibit limited proliferative and self-renewing capacities and are more prone to develop an exhausted phenotype. Indeed, old CAR-T cells under chronic stimulation in vitro had lower IFNγ and TNF production (Extended Data Fig. 1f,g), with no differences in the expression of exhaustion markers such as programmed cell death protein 1 (PD1), T cell immunoglobulin and mucin domain-containing protein 3 (TIM3), lymphocyte activation gene 3 (LAG3) or thymocyte selection-associated high-mobility group box protein (TOX) (Extended Data Fig. 1h–l). Altogether, these results indicate that age greatly influences the composition of CAR-T cell infusion products, favoring the accumulation of T_EM_ cells with higher cytotoxic properties but limited functions upon multiple rechallenges.

**Fig. 2:**
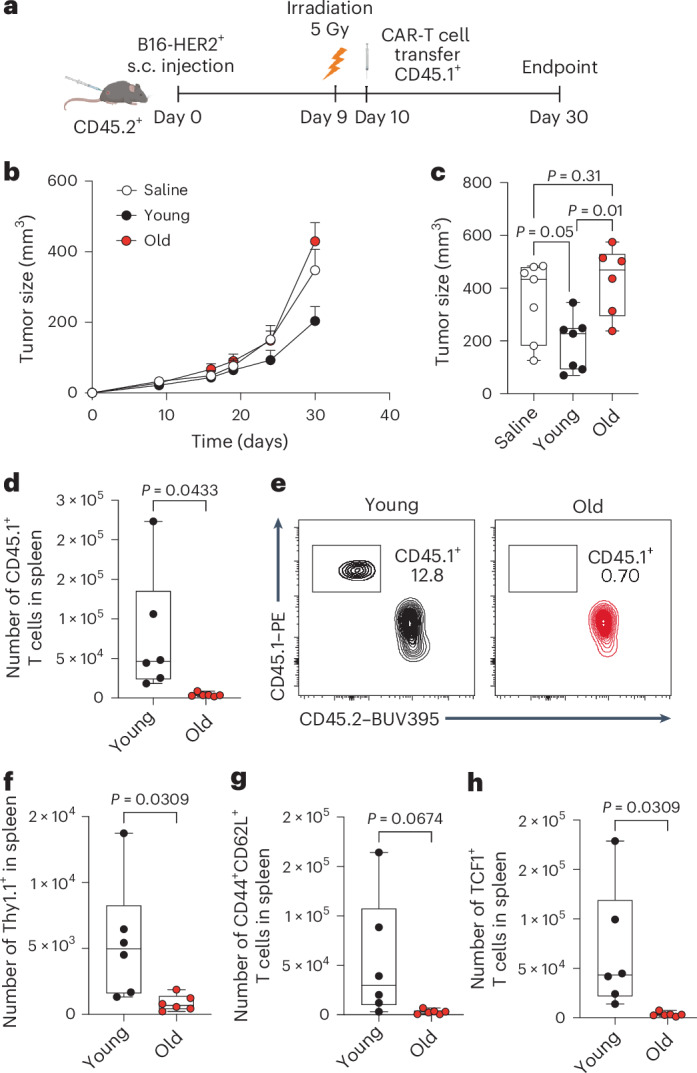
Age limits efficacy of CAR-T cell therapy. **a**, HER2-directed young or old CAR-T cells were adoptively transferred into mice bearing B16-HER2 tumors. Tumor growth was monitored and CAR-T persistence was determined on day 30. **b**, Follow-up of tumor growth over the course of the experiment. **c**, Tumor size (mm^3^) on day 30 after tumor engraftment (*n* = 7 saline, *n* = 7 young and *n* = 6 old). **d**, Number of adoptively transferred T cells (CD45.1^+^) in spleen after 30 days of tumor engraftment (*n* = 6 mice). **e**, Representative CD45.1/CD45.2 dot plots, gated within the CD8^+^ T cell populations in spleens. **f**–**h**, The number of CAR^+^ (Thy1.1^+^) (**f**), CD44^+^CD62L^+^ (**g**) and TCF1^+^ (**h**) T cells, gated within the CD3^+^CD8^+^CD45.1^+^CD45.2^−^ population (*n* = 6 mice). Data are presented as the mean values ± s.e.m. Statistical analysis was performed using a one-way ANOVA with multiple comparisons (**c**) or unpaired *t*-test (**d**,**f**–**h**), as appropriate. Panel **a** created with BioRender. Source data

**Extended Data Fig. 2:**
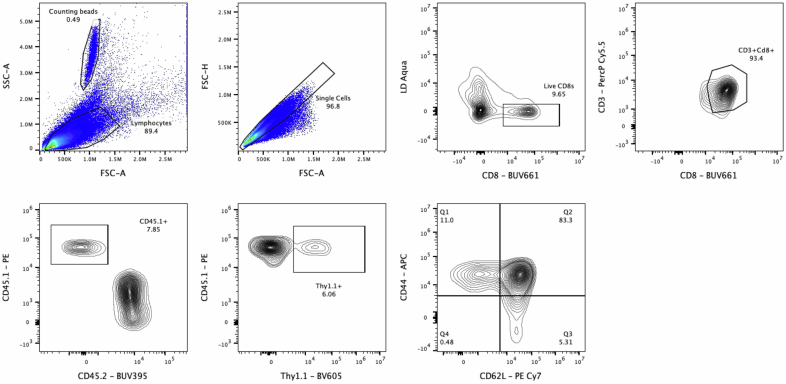
Gating strategy for murine CAR-T cells. Gating strategy, from top left to bottom right, to identify CD44^+^CD62L^+^ CAR-T cells in spleens after tumor engraftment. FSC-A/SSC-A: Lymphocytes > FSC-A/FSC-H: Single cells > LD^−^CD8^+^: Live CD8^+^ > CD3^+^CD8^+^: CD8^+^ lymphocytes > CD45.1^+^CD45.2^−^: Transferred cells > CD45.1^+^Thy1.1^+^: CAR-T cells > CD44^+^CD62L^+^: Tcm-like CAR-T cells. This gating strategy has been used for *in vivo* experiments included in Figs. 2 and 4i-n.

To determine whether aging would be a limiting factor for CAR-T cell efficacy, we next challenged the HER2-directed CAR-T cells in vivo (Fig. 2a). Specifically, we adoptively transferred young and old CAR-T cells (CD45.1^+^) into mice (CD45.2^+^) bearing HER2^+^ B16 tumors. We observed that only CAR-T cells derived from young mice and expanded with IL-7 and IL-15 but not those derived from aged mice could control tumor growth (Fig. 2b,c). No differences were observed between young and old CAR-T cells when expanded in IL-2 (Extended Data Fig. 1m). Young CAR-T cells are able to persist long-term in vivo, generating a pool of CD44^+^CD62L^+^TCF1^+^ CAR-T cells in the spleen that can potentially generate tumor-specific T_EM_ cells that can migrate to the tumor microenvironment (TME). Strikingly, the numbers of transferred (CD45.1^+^) and CAR-expressing (Thy1.1^+^) aged T cells were nearly undetectable 30 days after ACT in the spleen (Fig. 2d–f). Aged CAR-T cells completely lacked a memory or stem phenotype, as shown by the absence of CD44^+^CD62L^+^ (Fig. 2g) or TCF1^+^ T cells (Fig. 2h), underscoring their poor ability to persist in vivo, consistent with their T_EM_ commitment. Interestingly, aged CAR-T cells partially improved their persistence when transferred into older hosts, suggesting that age-dependent changes in the microenvironment can also influence CAR-T persistence (Extended Data Fig. 1n; gating strategy in Extended Data Fig. 2).

## NAD decline drives mitochondrial dysfunction in aged CAR-T cells

The development and maintenance of stem-like properties relies on the T cell capacity to boost mitochondrial metabolism^5,34,35^. Mitochondrial dysfunction has been extensively studied as a hallmark of aging^26,36^ but whether mitochondrial defects are the main driver of the loss of stemness in aged CD8^+^ T cells has not yet been elucidated.

**Fig. 3:**
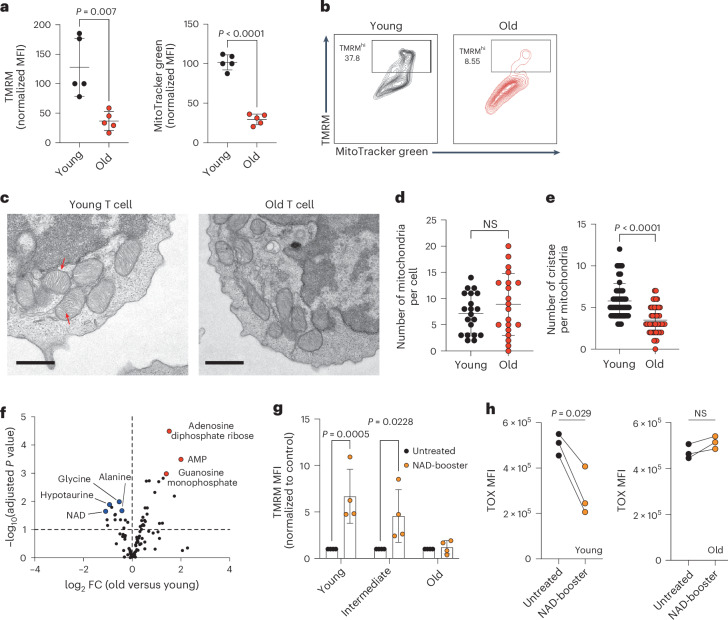
Aged CD8^+^ T cells present mitochondrial dysfunction associated with NAD decline. **a**, TMRM and MitoTracker green staining in freshly isolated bulk CD8^+^ T cells from the spleens of young and old mice. TMRM is a cell-permeable dye that accumulates in active mitochondria with intact membrane potential, while MitoTracker green binds to mitochondrial proteins giving a readout of mitochondrial activity and size, respectively (*n* = 5 biologically independent samples). **b**, Representative TMRM and MitoTracker green dot plots of data summarized in **a**. **c**, EM images of young and old naive CD8^+^ T cells. Red arrows indicate mitochondrial cristae. Scale bar, 1 μm. **d**,**e**, The number of mitochondria per cell (**d**) and the number of cristae per mitochondria (**e**) found by EM (*n* = 3 biologically independent samples). In **d**, dots represent the number of cells analyzed (*n* = 20). NS, not significant. In **e**, dots represent the number of mitochondria analyzed (*n* = 50). **f**, Volcano plot representing metabolomic data in young versus old CD8^+^ T cells (*n* = 5 biologically independent samples). **g**, Young (8 weeks old), intermediate (50 weeks old) and old (105 weeks old) CD8^+^ T cells were activated for 3 days and treated with the NAD precursor NMN (1 mM) for another 2 days, after which mitochondrial activity was assessed by TMRM staining (*n* = 4 biologically independent samples). **h**, Young and old CD8^+^ T cells were activated and expanded until day 7 in the presence of IL-7 and IL-15, after which they further received three rounds of CD3 restimulation every other day to promote an exhausted phenotype. Cells were treated with the NAD-booster NR (1 mM) and levels of the transcription factor TOX were determined on day 12 (*n* = 3 biologically independent samples). Data are presented as the mean values ± s.e.m. Statistical analysis was performed using an unpaired *t*-test (**a**,**d**,**e**), two-way ANOVA (**g**) or paired *t*-test (**h**), as appropriate. Statistical analysis of metabolomic data was performed using a two-way ANOVA on log_10_-transformed data and corrected with the Benjamini–Hochberg method. FC, fold change. Source data

**Extended Data Fig. 3:**
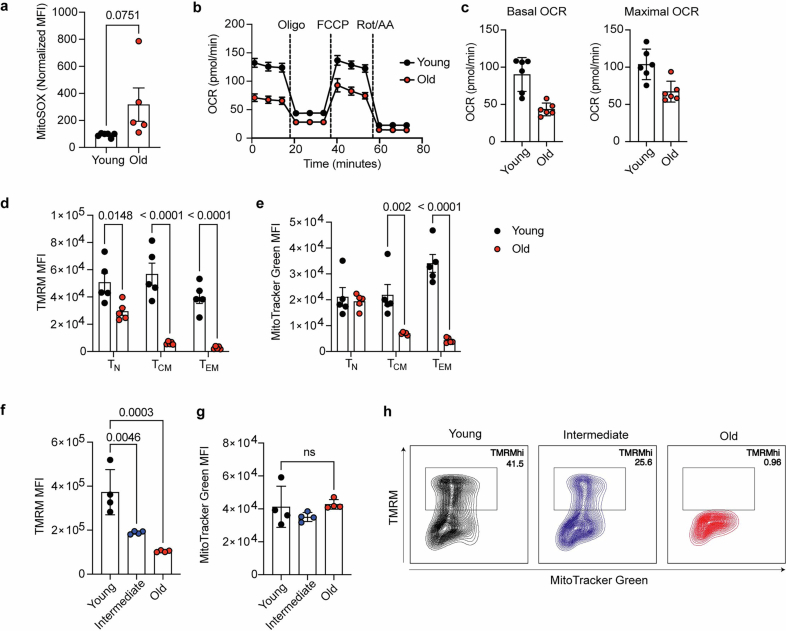
Aged CD8^+^ T cells present signs of mitochondrial dysfunction. (**a**) Mitochondrial ROS levels assessed by MitoSOX staining in young and old CD8^+^ T cells after 3 days of activation (n = 5 biologically independent samples). (**b**) OCR curve of young and old CD8^+^ T cells after 3 days of activation. In (**c**), basal OCR and maximal OCR values are represented (n = 3 biologically independent samples, in **C**, dots represent technical replicates from one representative experiment). (**d**) Mitochondrial membrane potential, as assessed by TMRM staining, of T_n_, T_cm_ and T_em_ populations gated within CD8^+^ T cells of splenocytes derived from young and old mice (n = 5 biologically independent samples). (**e**) Mitochondrial size, as assessed by MitoTracker Green staining, of T_n_, T_cm_ and T_em_ populations gated within CD8^+^ T cells of splenocytes derived from young and old mice (n = 5 biologically independent samples). Naïve CD8^+^ T cells (CD44^−^CD62L^+^) were isolated from splenocytes of young, intermediate and old mice and stained with TMRM (**f**) or MitoTracker Green (**g**) (n = 4 biologically independent samples). In (**h**), representative TMRM/MitoTracker Green dot plots are shown (n = 4 biologically independent samples). Data are represented as mean values +/- SEM. Statistical analysis was performed using unpaired *t*-test (**A** and **C**), two-way ANOVA (**D** and **E**), or one-way ANOVA with multiple comparisons (**F** and **G**), as appropiate. ns = p > 0.05. Source data

To this end, we first compared the mitochondrial profile of freshly isolated CD8^+^ T cells derived from the spleens of young and aged mice by examining mitochondrial membrane potential and mitochondrial mass by staining with tetramethylrhodamine (TMRM) and MitoTracker green, respectively. CD8^+^ T cells presented an age-dependent drop in both mitochondrial membrane potential and mass (Fig. 3a,b). Moreover, old CD8^+^ T cells accumulated mitochondrial reactive oxygen species (Extended Data Fig. 3a). Overall, these data suggest the acquisition of mitochondrial dysfunction in aged T cells, as previously reported^23,37–41^. Accordingly, old CD8^+^ T cells displayed a reduced basal and maximal oxygen consumption rate (OCR) upon T cell receptor stimulation (Extended Data Fig. 3b,c). To demonstrate that these mitochondrial deficiencies were cell-intrinsic defects induced by aging, we proved that mitochondrial activity was dampened in all T cell populations (naive T cell (T_N_), T_EM_ and T_CM_) (Extended Data Fig. 3d,e). Similarly, naive CD8^+^ T cells also presented an age-dependent progressive decrease in mitochondrial activity without altering mitochondrial size (Extended Data Fig. 3f–h). Electron microscopy (EM) confirmed these findings, as no differences were detected in the number or size of mitochondria (Fig. 3c,d) but aged naive CD8^+^ T cells had a reduced number of cristae (Fig. 3c,e), a feature of mitochondria with reduced activity.

To identify age-specific mechanisms driving mitochondrial dysfunction and CAR-T cell failure, we performed liquid chromatography with tandem mass spectrometry (LC–MS/MS) to analyze the metabolome of CD8^+^ T cells derived from young and old mice. Interestingly, aged CD8^+^ T cells had higher abundance for 15 metabolites, mostly related to purine and pyrimidine synthesis, such as adenosine diphosphate (ADP) riboside, adenosine monophosphate or guanosine monophosphate (Fig. 3f), and lower abundance for 11 metabolites when compared to young CD8^+^ T cells (Supplementary Table 1). Amongst the low-abundance metabolites, the levels of NAD were the most reduced (Fig. 3f). We and others have shown that supplementation with NAD precursors can increase NAD cellular levels and ameliorate mitochondrial function^42^. To this end, we treated aged and young T cells with the NAD precursor nicotinamide mononucleotide (NMN) and analyzed their mitochondrial activity. Interestingly, we found that, while NMN was able to improve the mitochondrial activity of young T cells, T cells derived from aged mice were irresponsive to NMN treatment (Fig. 3g). Similarly, old T cells treated with the NAD precursor nicotinamide riboside (NR), which we previously reported to prevent T cell exhaustion^23^, were not able to prevent TOX upregulation upon chronic in vitro stimulations, in contrast to younger counterparts (Fig. 3h). Overall, our findings demonstrate that aged CD8^+^ T cells accumulate important mitochondrial defects associated with NAD decline, which cannot be reversed through the administration of conventional NAD precursors.

## Restoration of NAD levels rescues functionality of aged CAR-T cells

**Fig. 4:**
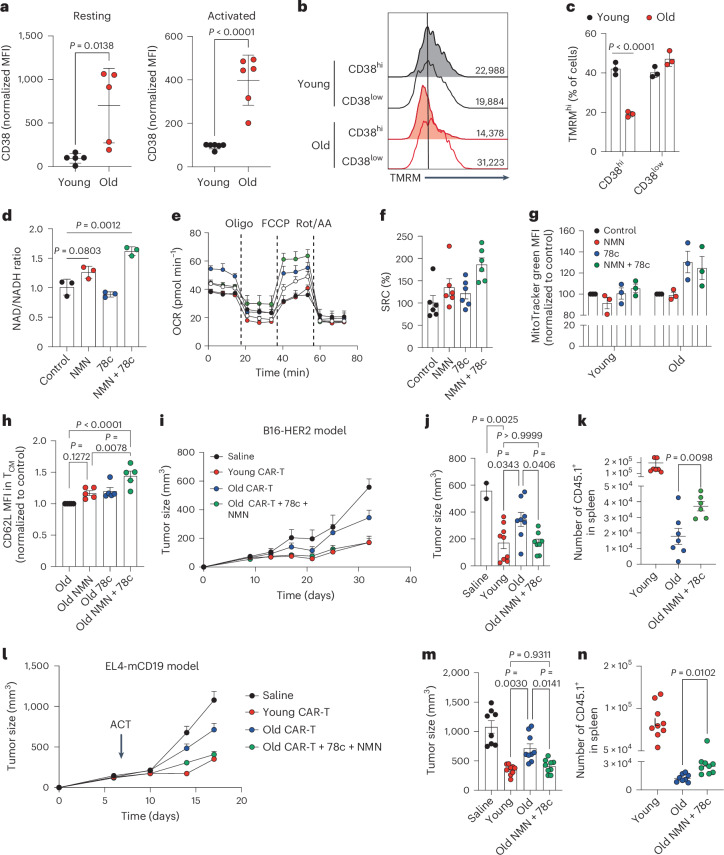
Restoration of NAD levels rescues functionality of aged CAR-T cells in vivo. **a**, Levels of CD38 in bulk CD8^+^ T cells upon isolation or 3 days after activation (*n* = 5 biologically independent samples). **b**, Representative TMRM histograms of CD38^hi^ and CD38^low^ population in young and old CD8^+^ T cells 3 days after activation. **c**, Proportion of TMRM^hi^ cells in CD38^hi^ and CD38^low^ populations (*n* = 3 biologically independent samples). **d**, NAD/NADH ratio on day 7 in old CAR-T cells expanded with IL-7 and IL-15 and treated with NMN and/or 78c, a specific inhibitor of the NADase enzymatic activity of CD38 (*n* = 3 biologically independent samples). **e**, OCR on day 7 in old CAR-T cells expanded with IL-7 and IL-15 and treated with NMN and/or 78c were measured using a Seahorse XFe96 Analyzer. During this assay, mitochondrial fitness was assessed upon the sequential addition of oligomycin (oligo, adenosine triphosphate synthetase inhibitor), FCCP (mitochondrial membrane uncoupler) and rotenone + antimycin A (Rot/AA, complex I and III inhibitors, respectively). **f**,**g**, Further analysis of the recovery of SRC (**f**) and mitochondrial size (**g**) of old CAR-T cells treated with NMN and 78c. Seahorse data displayed in **f** are representative of three independent experiments (*n* = 6 technical replicates). For **g**, *n* = 3 biologically independent samples. **h**, CD62L levels within T_CM_ populations (*n* = 5 biologically independent samples). In **i**–**k**, young and old CAR-T cells treated with or without NMN and 78c were adoptively transferred into mice bearing B16-HER2 tumors. **i**,**j**, Tumor growth (**i**) and tumor size at last time point (day 30) (**j**) (*n* = 9 young, n = 8 old and *n* = 8 old 78c + NMN). **k**, Number of transferred T cells (CD45.1^+^) found in spleen 32 days after tumor engraftment (*n* = 7 mice). In **l**–**n**, young and old CAR-T cells treated with or without NMN and 78c were adoptively transferred into mice bearing EL4-mCD19 tumors (*n* = 8 mice per group). **l**,**m**, Tumor growth (**l**) and tumor size at last time point (day 17) (**m**). **n**, Number of transferred T cells (CD45.1^+^) found in spleen 17 days after tumor engraftment. Data are presented as the mean values ± s.e.m. Statistical analysis was performed using an unpaired *t*-test (**a**,**k**,**n**), two-way ANOVA (**c**) or one-way ANOVA with multiple comparisons (**d**–**m**), as appropriate. Source data

**Extended Data Fig. 4:**
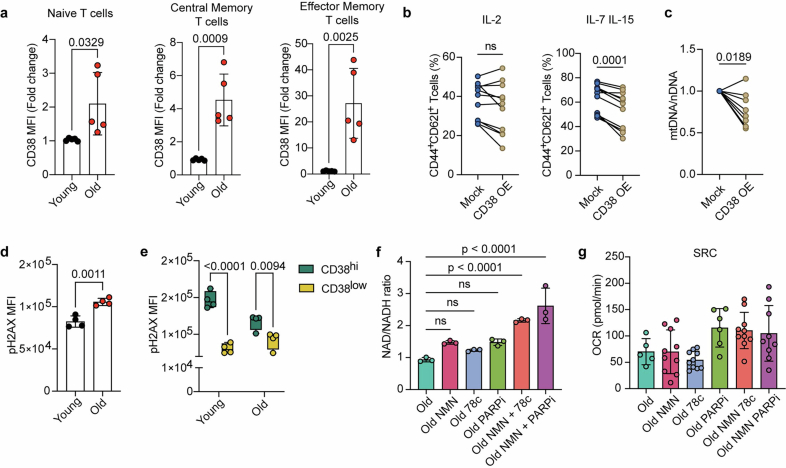
NAD metabolism is altered in aged CD8^+^ T cells. (**a**) Levels of CD38 within T_n_, T_cm_ and T_em_ populations in young and old CD8^+^ T cells isolated from splenocytes (n = 5 biologically independent samples). (**b**) Proportion of T_cm_ cells on day 7 upon activation of naïve CD8^+^ T cells derived from young mice (8 weeks old) transduced with a CD38 OE vector and expanded under effector (IL-2) or memory (IL-7 IL-15) polarizing conditions (n = 9 biologically independent samples). (**c**) mtDNA/nDNA ratio in young CD8 + T cells overexpressing CD38 after 7 days of expansion with IL-7 and IL-15 (n = 9 biologically independent samples). (**d**) DNA damage, as assessed by pH2AX staining, in freshly isolated CD8^+^ T cells from young and old mice (n = 4 biologically independent samples). (**e**) pH2AX levels in CD38^hi^ and CD38^low^ CD8^+^ T cells from young and old mice (n = 4 biologically independent samples). HER2-directed CAR-T cells were generated from old mice and expanded in the presence of IL-7 and IL-15 ± NMN ± 78c ± Olaparib (PARPi). On day 7, NAD/NADH ratio (**f**) and SRC as assessed by Seahorse XFe96 Analysis (**g**) were determined (n = 3, in (**G**) dots represent technical replicates from one representative experiment). Data are represented as mean values +/- SEM. Statistical analysis was performed using unpaired *t*-test (**A** and **D**), paired *t*-test (**B** and **C**), two-way ANOVA (**E**), or one-way ANOVA with multiple comparisons (**F** and **G**), as appropiate. ns = p > 0.05. Source data

An investigation by Camacho-Pereira et al. revealed that one of the main triggering factors of NAD decline in WAT, muscle and liver during aging is the systemic upregulation of CD38 (ref. ^27^). CD38 is a multifunctional enzyme that degrades NAD and modulates NAD homeostasis in a variety of immune cells^43,44^. CD38-mediated NAD-degradation leads to the production of substrates (namely, cyclic ADP-ribose and nicotinic acid adenine dinucleotide phosphate) that are subsequently involved in the regulation of Ca^2+^ signaling and the production of adenosine, an important metabolite with immunosuppressive functions^43,44^. Moreover, it has been reported that CD38 can also mediate the degradation of NAD precursors, including NMN and NR^45^. Thus, we tested whether CD38 could impact aged CD8^+^ T cells function by (1) limiting NAD metabolism and mitochondrial activity and (2) preventing responsiveness to NAD precursors. We investigated how CD38 levels change during T cell aging and we found that CD38 is expressed to a higher level in old CD8^+^ T cells, in both resting and activated states (Fig. 4a). Importantly, this phenomenon was observed in all CD8^+^ T cell populations (T_N_, T_EM_ and T_CM_) (Extended Data Fig. 4a). To determine whether CD38 is a limiting factor of mitochondrial activity in aged CD8^+^ T cells, we measured mitochondrial membrane potential in T cell subpopulations with high (CD38^hi^) and low (CD38^low^) CD38 expression. Importantly, we found that, in aged CD8^+^ T cells but not in younger controls, only CD38^hi^ T cells displayed reduced mitochondrial activity (Fig. 4b,c). Moreover, young CD8^+^ T cells transduced with a CD38-overexpressing construct exhibited a limited generation of T_CM_ cells and decreased mitochondrial DNA (mtDNA) content (Extended Data Fig. 4b,c), recapitulating the features of aged CD8^+^ T cells. These results suggest that CD38 can modulate the mitochondrial activity and fate decision of CD8^+^ T cells, creating a link among CD38, aging, mitochondrial fitness and maintenance of T cell stemness.

However, CD38 is not the only enzyme impacting NAD cellular levels in aging. For instance, alterations in NAD synthesis pathways (that is, the salvage or de novo synthesis pathways) or the activation of NAD-consuming enzymes can strongly modulate NAD cellular levels^28^. In particular, poly(ADP-ribose) polymerases (PARPs) have been described as the major NAD-consuming enzymes in the cell^29,46^, where their activation has often been associated with aging because of the accumulation of age-dependent DNA damage. However, it is not known whether this phenomenon is present in aged CD8^+^ T cells and can affect NAD levels and mitochondrial metabolism. To this end, we first measured DNA damage by analyzing the phosphorylation levels of γH2AX and found that aged CD8^+^ T cells exhibited a significant increase (Extended Data Fig. 4d). Moreover, we identified that CD38^hi^ T cells, that is, bearing defective mitochondria (Fig. 4b), accumulated more p-γH2AX than CD38^low^ T cells (Extended Data Fig. 4e). These results suggest that the mitochondrial defects in aged CD8^+^ T cells derive from a multifactorial process impacting NAD homeostasis.

Next, we aimed to elucidate whether restoration of NAD levels is sufficient to reinstate mitochondrial fitness and functionality of aged CAR-T cells. To this end, we used the small molecule 78c to specifically block the NADase activity of CD38 (ref. ^47^) and we combined it with NMN supplementation. We observed that, according to the previous data (Fig. 3g,h), NMN alone was not sufficient to increase NAD levels in aged CD8^+^ T cells (Fig. 4d). However, when combined with the CD38 inhibitor 78c, NAD levels were restored to levels seen in younger controls (Fig. 4d). Consequently, 78c + NMN supplementation during ex vivo expansion of CAR-T cells repaired the mitochondrial function of aged CAR-T cells, as shown by an increased spare respiratory capacity (SRC) and mitochondrial mass (Fig. 4e–g). Importantly, the effects of the 78c + NMN treatment were observed in aged T cells but not in younger controls, suggesting that this combination strategy is particularly beneficial in contexts where CD38 levels are aberrantly high. Furthermore, combining 78c and NMN improved the phenotype of the CAR-T infusion products, as shown by a heightened expression of CD62L within the T_CM_ population (Fig. 4h). Similarly, inhibition of PARP activity using olaparib also rescued the NAD levels and mitochondrial activity of aged CD8^+^ T cells (Extended Data Fig. 4f,g).

**Extended Data Fig. 5:**
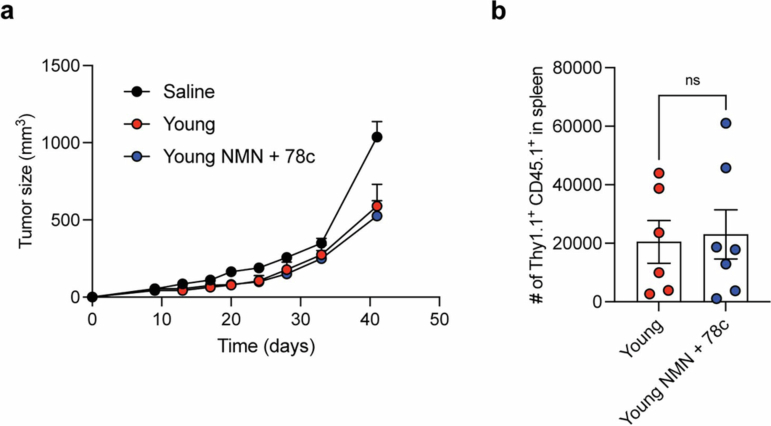
Modulation of NAD levels does not improve efficacy of young CAR-T cells. Her2-directed CAR-T cells were generated using isolated CD8^+^ T cells from young mice (8 weeks old), expanded in the presence of IL-7 and IL-15, with or without NMN and 78c and adoptively transferred at day 10 into mice bearing B16-HER2^+^ tumors. Tumor growth is displayed in (**a**), while number of transferred CAR-T cells (CD45.1^+^Thy1.1^+^) found in spleen after 41 days post-tumor engraftment is displayed in (**b**) (n = 6 Young, n = 7 Young NMN + 78c). Data are represented as mean values +/- SEM. Statistical analysis was performed using unpaired *t*-test. ns = p > 0.05. Source data

To assess whether the recovery of the mitochondrial fitness was sufficient to restore CAR-T cell functionality in vivo, we reinfused young and old CAR-T cells with or without 78c + NMN treatment in mice bearing HER2^+^ B16 tumors. We observed that, whereas the untreated aged CAR-T cells were unable to control tumor growth, aged CAR-T cells supplemented with 78c + NMN efficiently controlled tumor growth in line with young CAR-T cells (Fig. 4i,j). Importantly, treated aged CAR-T cells prolonged their long-term survival in vivo, as shown by a significant increase in the number of CD44^+^CD62L^+^TCF1^+^ CAR-T cells found in spleens 30 days after ACT (Fig. 4k). Supplementing young CAR-T cells with NMN + 78c during the ex vivo expansion did not result in improved tumor growth control (Extended Data Fig. 5a) or increased persistence in vivo (Extended Data Fig. 5b), indicating that NMN + 78c treatment restores CAR-T cell functionality specifically in the context of aging.

**Extended Data Fig. 6:**
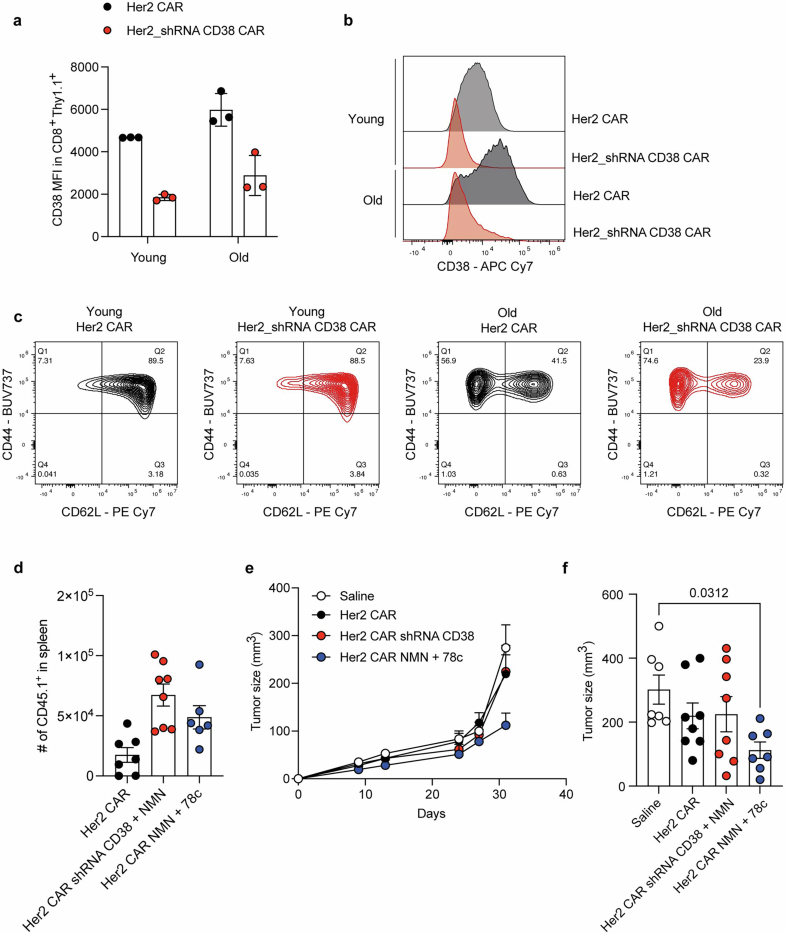
CD38 downregulation does not improve functionality of aged CAR-T cells. Young and old CD8^+^ T cells were activated and transduced with an Her2 CAR or an Her2 CAR vector modified to contain an shRNA for CD38. On day 3, after transduction, CAR-T cells were expanded in the presence of IL-7 and IL-15 until day 7. In (**a**), protein levels of CD38 as assessed by flow cytometry on day 7. A representative histogram is shown in (**b**) (n = 3 biologically independent samples). (**c**) CD44/CD62L plots of young and old Her2 CAR T cells (Ctrl vs shRNA CD38) at day 7 of expansion. For (**d**), (**e**) and (**f**), old Her2 CAR-T cells, old Her2_shRNA CD38 CAR-T cells (expanded in the presence of NMN) or old Her2 CAR-T cells expanded in the presence of NMN + 78c were adoptively transferred at day 10 into mice bearing B16-HER2 tumors (n = 8 per group). Number of transferred CAR-T cells (CD45.1^+^) found in spleen after 32 days post-tumor engraftment is displayed in (**D**). Tumor growth is displayed in (**E**), while tumor size at endpoint is shown in (**F**). Data are represented as mean values +/- SEM. Statistical analysis was performed using one-way ANOVA. Source data

Additionally, we modified the HER2 CAR construct to integrate a short hairpin RNA (shRNA) for CD38 (hereafter referred as HER2 CAR_shRNA CD38) as a strategy to modulate CD38 enzymatic activity by reducing its expression. Upon transduction, both young and old CD8^+^ T cells presented a highly significant downregulation of CD38 protein levels (Extended Data Fig. 6a,b). HER2 CAR_shRNA CD38 T cells generated from young CD8^+^ T cells and expanded for 7 days in the presence of IL-7 and IL-15 showed a similar T_CM_ phenotype. Interestingly, downregulation of CD38 levels in old CAR-T cells resulted in a worsened expansion of T_CM_ cells, even when expanded in the presence of NMN (Extended Data Fig. 6c). To assess their functionality, we transferred either old CAR_shRNA CD38 T cells, old CAR_shRNA CD38 T cells expanded with NMN or old control CAR-T cells expanded with NMN + 78c into mice bearing HER2^+^ B16 tumors. CD38 downregulation led to an increased number of CAR-T cells in the spleens 30 days after ACT (Extended Data Fig. 6d). However, the control CAR-T cells supplemented with NMN + 78c but not old CAR_shRNA CD38 T cells were able to successfully control tumor growth (Extended Data Fig. 6e,f). These results underline the importance of specifically targeting the NADase activity of CD38 and suggest that the recovery of CAR-T cell functionality through CD38 is NAD dependent.

To validate our findings in alternative CAR-T cell models, we extended our study using anti-CD19_28z (ref. ^48^), which targets a different antigen and contains a different costimulatory domain. CD19-directed CAR-T cells were generated from young and old CD8^+^ T cells (CD45.1^+^) and were adoptively transferred into mice (CD45.2^+^) engrafted with EL4 lymphoma cancer cells overexpressing mouse CD19 (mCD19) (Fig. 4l). Tumor control capacity was monitored over time. Similarly to the B16-HER2 model, young CAR-T cells were able to successfully control tumor growth, whereas old CAR-T cells failed (Fig. 4m). Importantly, old CAR-T cells expanded in the presence of NMN and 78c recovered their functionality in vivo, as shown by improved tumor growth control (Fig. 4m). Analysis of the spleens at endpoint (day 17) further revealed an increased number of CD45.1^+^ T cells, indicating improved persistence (Fig. 4n).

Overall, these data demonstrates that NAD metabolism, which is greatly compromised during aging, is a key determinant of CAR-T cell successful responses. Combined strategies to boost NAD levels, such as NAD precursors together with a CD38 or PARP inhibitor, rejuvenates aged CAR-T cells, ultimately leading to improved therapeutic efficacy.

## Aging and NAD metabolism determine responses to CAR-T cell therapy

**Fig. 5:**
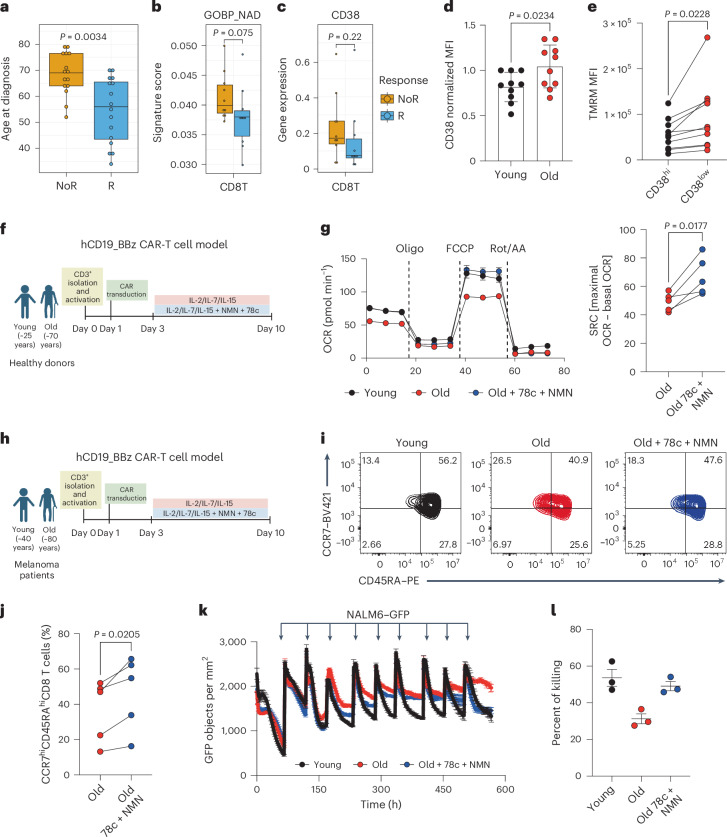
Age and NAD metabolism determine responses to CAR-T cell therapy in persons with cancer. **a**, Age of the participants at the time of diagnosis of R (*n* = 16) and NoR (*n* = 15) participants with LBCL to anti-CD19 CAR-T cell therapy. **b**,**c**, NAD metabolism (Gene Ontology: 0019674) signature score (**b**) and CD38 expression levels (**c**) in baseline PBMCs from R (*n* = 10) and NoR (*n* = 10) participants to anti-CD19 CAR-T cell therapy. Data included in **a**–**c** were taken from recently published scRNA-seq data from Haradhvala et al. (2022) (GSE197268)^49^. **d**, CD38 levels in CD8^+^ T cells from PBMCs derived from young and old participants with melanoma (*n* = 10). **e**, Mitochondrial activity in CD38^hi^ and CD38^low^ populations of CD8^+^ T cells from PBMCs derived from participants with melanoma, as assessed by TMRM staining (*n* = 10). **f**, Schematic representation of experimental setup to target NAD metabolism in human anti-CD19 CAR-T cells generated from young (<28 years old) and elderly (>66 years old) healthy donors. **g**, OCR curve of young and old CAR-T cells with or without NMN and 78c treatment analyzed on day 10 with Seahorse XFe96. Right: further analysis of the recovery of SRC of old CAR-T cells treated with NMN and 78c (*n* = 5 healthy donors). In **h**–**l**, PBMCs from young and old participants with melanoma were used to generate hCD19_BBz CAR-T cells, which were expanded for 10 days in the presence of IL-2, IL-7 and IL-15 with or without 78c and NMN treatment. **h**, Schematic representation of experimental setup to target NAD metabolism in human anti-CD19 CAR-T cells generated from young (~40 years old) and elderly (~80 years old) participants with melanoma. **i**, Representative CCR7 and CD45RA dot plots, as assessed by flow cytometry. **j**, Further analysis of the recovery of CCR7^hi^CD45RA^hi^ cells of old CAR-T cells treated with NMN and 78c (*n* = 5 participants). **k**, hCD19-BBz CAR-T cells were cocultured with NALM6–GFP cells in a 1:4 effector-to-target ratio to determine killing capacity using Incucyte. Arrows represent rechallenge of CAR-T cells with NALM6–GFP cells every 2–3 days (*n* = 2). **l**, Quantification of the percentage killing at the last time point. Here dots represent technical replicates from one of two independent experiments. Data are presented as the mean values ± s.e.m. Statistical analysis was performed using an unpaired *t*-test (**a**–**d**), paired *t*-test (**e**,**g**,**j**) or one-way ANOVA (**l**), as appropriate. Panels **f**, **h** created with BioRender. Source data

**Extended Data Fig. 7:**
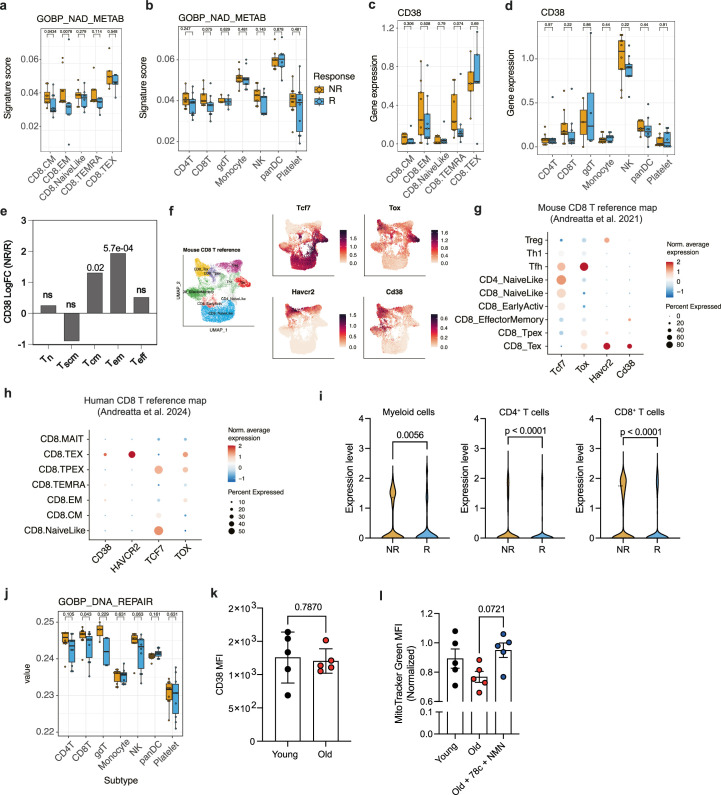
NAD metabolic pathways in human CD8^+^ T cells and CAR-T cell responses. NAD metabolism signature score in CD8^+^ T cell subtypes (**a**) and other immune cells (**b**) from baseline PBMCs of responder and non-responder patients to anti-CD19 CAR-T cell therapy. Analysis was performed using the UCell method^68^ on recently published scRNAseq data from Haradhvala et al (2022)^49^. From the same dataset, CD38 expression levels in CD8^+^ T cell subsets and other immune cells are shown in (**c**) and (**d**), respectively (n = 15 R, n = 16 NoR). (**e**) Comparison of CD38 levels in baseline PBMCs of B cell malignancies patients presenting long-term ( > 6 months) vs short-term ( < 6 months) CAR-T cell persistence upon infusion, using publicly available RNAseq data from Chen et al (2021) (n = 71 patients)^50^. (**f-g**) Expression levels of *Tcf7*, *Tox*, *Havcr2* and *Cd38* on a reference map of mouse CD8 T cells, in UMAP space (**F**) and averaged by T cell subtype (**G**). (**h**) Comparison of *TCF7*, *TOX*, *HAVCR2* and *CD38* expression levels in human CD8 T cell subsets^66^. (**i**) *CD38* expression levels in myeloid cell, CD4^+^ T cells and CD8^+^ T cells of tumor immune infiltrates of responder (n = 17) and non-responder (n = 31) patients to ICB^51^. (**j**) DNA damage signature score in immune cells from baseline PBMCs of responder and non-responder patients to anti-CD19 CAR-T cell therapy (n = 10 patients)^49^. (**k**) CD38 protein levels in CD4^+^ T cells analyzed in PBMCs derived from young (39-49 years old) and old (76-84 years old) melanoma patients (n = 5) (**l**) Mitochondrial size, as assessed by MitoTracker Green staining, of hCD19_BBz CAR-T cells generated from PBMCs derived from young and old healthy donors and expanded in the presence of IL-2, IL-7 and IL-15 + /- NMN and 78c. Data are represented as mean values +/- SEM. Statistical analyses were performed using unpaired *t*-test or one-way ANOVA, as appropiate. Source data

To assess the influence of aging and NAD metabolism on CAR-T clinical application, we reanalyzed recently published anti-CD19 CAR-T data from 31 participants with large B cell lymphoma (LBCL) (15 responders (R) and 16 nonresponders (NoR))^49^. Importantly, we found that older participants had inferior responses (Fig. 5a). Moreover, single-cell transcriptomic analysis on baseline peripheral blood mononuclear cells (PBMCs) of 20 participants with LBCL (10 R and 10 NoR) revealed differences in NAD metabolic signatures of CD8^+^ T cells from R and NoR participants (Fig. 5b and Extended Data Fig. 7a). These differences were also detected in natural killer (NK) cells but not in other immune cells (Extended Data Fig. 7b).

Following our previous analysis on NAD-related pathways, we focused on elucidating how CD38 is involved in CAR-T cell outcomes. We found that CD8^+^ T cells from NoR participants presented a tendency to express higher *Cd38* baseline levels (Fig. 5c and Extended Data Fig. 7c). Similarly to the NAD signature, *Cd38* levels were also lower in the NK cells of R participants (Extended Data Fig. 7d). In addition, using a transcriptomic atlas (https://tanlab4generegulation.shiyapps.io/Tcell_Atlas/)^50^ of premanufactured T cells from 71 participants with B-ALL, we found that increased *Cd38* baseline levels across several T cell subtypes dampened CAR-T cell persistence upon infusion (Extended Data Fig. 6e), suggesting that CD38 is associated with the stemness potential of CAR-T cells. Previous investigations characterized CD38 as a marker of terminally exhausted T cells in both murine (Extended Data Fig. 7f,g) and human (Extended Data Fig. 7h) settings. Consistently, TILs of NoR participants to immune checkpoint blockade (ICB) accumulate CD8^+^ T cell clusters that present an exhausted signature and higher *Cd38* levels^51^, a phenomenon also observed in CD4^+^ T cells and myeloid cells (Extended Data Fig. 7i). Our findings, however, add to CD38 a role as a non-exhaustion-related predictive marker of CAR-T cell persistence and efficacy. Of note, other NAD-associated pathways such as DNA damage were also predictive of the response (Extended Data Fig. 7j) suggesting that general NAD signatures in baseline PBMCs could be used to predict CAR-T cell responses.

To test whether NAD metabolism could also be targeted to rejuvenate human CAR-T cells, we first analyzed PBMCs derived from participants with melanoma and found an age-dependent increased expression of CD38 in CD8^+^ T cells (Fig. 5d) but not in CD4^+^ T cells (Extended Data Fig. 7k). To determine whether CD38 expression was altering mitochondrial activity, we compared TMRM staining in CD38^hi^ and CD38^low^ CD8^+^ T cells and observed that mitochondrial potential was particularly restricted in those cells expressing higher levels of CD38 (Fig. 5e).

Next, we generated human CD19 CAR-T cells containing a 41BB costimulatory domain using PBMCs derived from young (<30 years old) and old (>65 years old) healthy donors, expanded them in the presence of IL-2, IL-7 and IL-15 and compared their mitochondrial fitness (Fig. 5f). CAR-T cells generated from older donors displayed a significant reduction in SRC (Fig. 5g) and mitochondrial mass (Extended Data Fig. 7l). Remarkably, old CAR-T cells supplemented with 78c and NMN during ex vivo expansion were able to revert their mitochondrial defects and reestablish a mitochondrial profile of young CAR-T cells (Fig. 5g and Extended Data Fig. 7l). These results suggest that age-associated metabolic defects in CAR-T cells are also observed in a human setting and can be reverted by modulating NAD levels.

**Extended Data Fig. 8:**
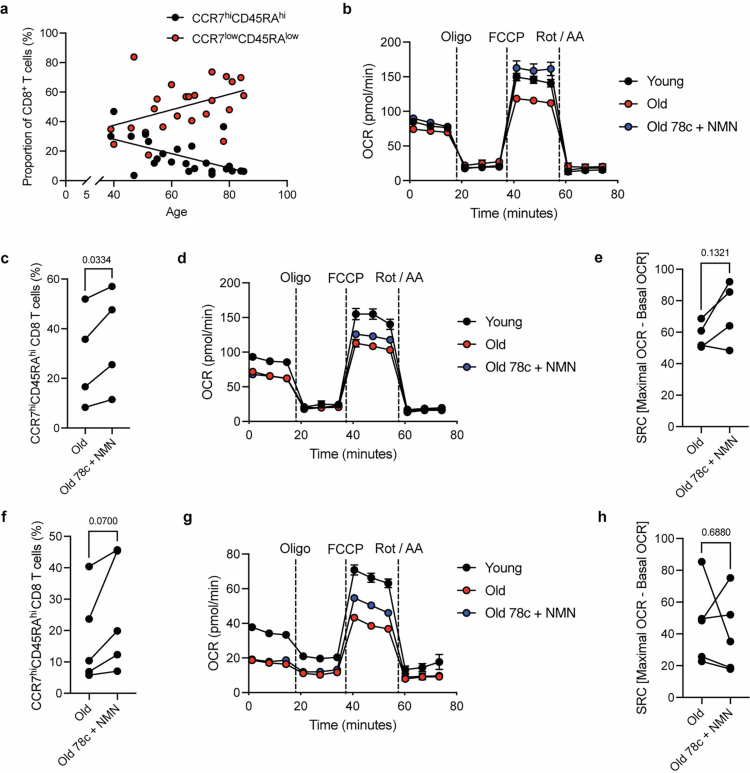
Restoration of NAD metabolism improves CAR-T cell function and metabolism. (**a**) Phenotype of PBMCs derived from young and old melanoma and non-small cell lung cancer (NSCLC) was assessed by flow cytometry. Proportion of CCR7^hi^CD45RA^hi^ / CCR7^low^CD45RA^low^ cells within CD8^+^ T cells is displayed (n = 24 patients). (**b**) hCD19_BBz CAR-T cells were generated from young (39-49 years old) and old (76-84 years old) melanoma patients PBMCs and expanded using IL-2, IL-7 and IL-15. On day 10, mitochondrial activity was assessed using Seahorse XFe96 Analyzer (n = 3 patients). (**C**-**E**) hCD19_28z CAR-T cells were generated from young (39-49 years old) and old (76-84 years old) melanoma patients and expanded using IL-2, IL-7 and IL-15 (n = 4 patients) (**c**) The proportion of CCR7^hi^CD45RA^hi^ CAR-T cells within the CD8^+^ T cell population was assessed by flow cytometry. (**d**) Mitochondrial activity was assessed performing a MitoStress Test with Seahorse XFe96 Analyzer. (**e**) SRC of control and treated (78c + NMN) old CAR-T cells. (F-H) hCD19_BBz CAR-T cells were generated from young (39-49 years old) and old (76-84 years old) melanoma patients PBMCs and expanded using IL-2 only (n = 5 patients) (**f**) The proportion of CCR7^hi^CD45RA^hi^ CAR-T cells within the CD8^+^ T cell population was assessed by flow cytometry. (**g**) Mitochondrial activity was assessed performing a MitoStress Test with Seahorse XFe96 Analyzer. (**h**) SRC of control and treated (78c + NMN) old CAR-T cells. Data are represented as mean values +/- SEM. Statistical analyses were performed using paired *t*-test. Source data

To further investigate the importance of aging and NAD metabolism in a clinical setting, we analyzed PBMCs derived from participants with melanoma and non-small cell lung cancer. We showed that older participants gradually accumulate C–C chemokine receptor type 7 (CCR7)^low^CD45RA^low^ T cells while losing CCR7^hi^CD45RA^hi^ T cells (Extended Data Fig. 8a). To test whether these phenotypic differences could also be observed by the end of the expansion of CAR-T cells, we generated human CAR-T cells using PBMCs from young and old participants with melanoma using the same hCD19_BBz model (Fig. 5h). We analyzed the phenotype of CAR-T cells and indeed observed that old CAR-T cells had a lower proportion of CCR7^hi^CD45RA^hi^ T cells when compared to younger controls. Treatment with NMN + 78c was able to increase the proportion of this T cell subset (Fig. 5i,j). Similarly to the CAR-T cells generated from healthy donors, CAR-T cells from older participants with melanoma presented an impaired mitochondrial function that was reverted upon expansion with NMN + 78c (Extended Data Fig. 8b). Of note, these differences were also observed in a human CD19 CAR-T cell model containing a CD28 costimulatory domain (Extended Data Fig. 8c–e). Old hCD19_BBz CAR-T cells expanded using only IL-2 did not present metabolic recovery upon NMN + 78c supplementation, while showing an increased proportion of CCR7^hi^CD45RA^hi^ cells (Extended Data Fig. 8f–h).

Lastly, to investigate whether the metabolic and phenotypic changes translated into recovery of the functionality of human CAR-T cells, we cocultured CAR-T cells with NALM6–GFP and tracked their killing capacity upon multiple rechallenges performed every 2–3 days. We observed few differences in killing capacity between young and old CAR-T cells at early stages (Fig. 5k,l). However, old CAR-T cells lost their tumor killing capacity upon multiple rechallenges, whereas young CAR-T cells were able to maintain it. Importantly, old human CAR-T cells treated with NMN + 78c recovered their functionality, as shown by a prolonged maintenance of killing capacity upon multiple rechallenges (Fig. 5k,l). Overall, these data indicate that age determines the phenotype, functionality and metabolic status of CAR-T cells and manipulating NAD metabolism can be used as a strategy to boost their function.

Altogether, our data demonstrate that the age-dependent NAD decline leads to mitochondrial defects and loss of stem-like properties in T cells, ultimately resulting in CAR-T cell failure. Moreover, we establish the restoration of NAD cellular levels as a strategy to recover mitochondrial function and rejuvenate CAR-T cells in the context of aging.

## Discussion

Several drivers of aging (for example, genomic instability, epigenetic alterations, chronic inflammation or mitochondrial dysfunction) are also common drivers of tumorigenesis, which make aging the first risk factor associated with cancer incidence^52^. In this context, the field of immuno-oncology has greatly expanded during the last decade; however, few studies have investigated how aging impacts immunotherapy efficacy. In the context of ICB, data acquired from preclinical models and clinical trials are currently unclear and contradictory^53^. In mice, some studies have shown decreased response to anti-PD1 or anti-cytotoxic T lymphocyte-associated protein 4 therapy in aged mice^54,55^, while other studies have documented an intact or even superior response to PD1 and its ligands^56^. These discrepancies might be explained by the fact that the expression of PD1 and its ligands is altered during aging in a cell-specific and organ-specific manner and aging affects mutagenesis burden in the tumor, thus influencing T cell infiltration in the TME^57^. Overall, in ICB, there are several factors independent of the intrinsic effect of aging on T cells that can determine the response to therapy. Indeed, although it is just beginning to be appreciated^24^, to date, no evidence supports the notion that aging might be an important limiting factor of CAR-T cell therapy efficacy in the clinic. In this Article, we demonstrated that CAR-T cells generated from aged mice display qualitative defects associated with an inability to maintain stem-like properties. We show that the lack of stem-like properties arises from mitochondrial dysfunction derived from the decline in NAD cellular levels and its recovery is sufficient to rejuvenate the functionality of aged CAR-T cells. Moreover, we provide clinically relevant data showing that NAD metabolic pathways can be both targeted and used as predictive markers of CAR-T cell therapy efficacy.

The role of NAD metabolism in T cell function and antitumor responses has been widely explored. A report from Chatterjee et al. (2018) described a potent antitumor T helper 1 and 17 hybrid cell that was able to maintain effector functions while persisting long-term in vivo^21^. Interestingly, these cells were dependent on a higher NAD-dependent activity of the histone deacetylase SIRT1 (ref. ^21^). Other studies have also applied NAD-boosting strategies to prevent T cell exhaustion^23^ or ameliorate CAR-T cell and TIL therapy^20,58^, providing promising results in preclinical models. As a result, the use of NAD precursors, such as NR or NMN, is gaining relevance in the field of immunotherapy. However, a limitation of current preclinical studies is the lack of aged mice included within the experimental design, which might lead to real-world discrepancies and difficulties to move from bench to bedside. For example, the administration of NAD precursors as a nutritional supplement has been included in several clinical trials to treat cardiovascular, neurodegenerative and metabolic diseases but their efficacy has been limited when applied in older subjects. In our study, we found that the sole administration of NAD precursors is indeed insufficient to improve the fitness of aged cells. These findings emphasize the need to find alternative or complementary strategies to increase NAD cellular levels and benefit from its effects. For CD8^+^ T cells, we explored the CD38 inhibitor 78c in combination with NAD precursors but we cannot exclude other strategies based on additional mechanisms of NAD homeostasis disturbance (PARP inhibitors in combination with NAD precursors). Nonetheless, not all cell types upregulate CD38 and might benefit from their strategy. Other immune cells, such as macrophages, present decreased activity of quinolinate and nicotinamide phosphoribosyltransferases during aging, both involved in NAD synthesis pathways, and recovery of their activity reinstates macrophage functionality^59,60^. These results show the importance of maintaining NAD homeostasis during aging for optimal immune function and suggest a tailored NAD-boosting strategy depending on the cell type and context.

Although we focused on the intrinsic defects of CAR-T cells during aging in this study, important factors to consider are the extrinsic barriers that might impede CAR-T cell efficacy. Aging also fosters an environment marked by the presence of senescent cells and systemic low-grade chronic inflammation, known as ‘inflammaging’. Several investigations have described changes in the composition of the TME with aging, including an accumulation of fibroblasts and immunosuppressive cell subsets associated with a senescence-associated secretory phenotype such as myeloid-derived suppressor cells and regulatory T cells. However, whether tumor initiation and progression are aggravated during aging is still unclear, as some reports have described faster tumor growth in aged mice while other studies support the opposite. Similarly, it is widely discussed whether senescence might have protumoral or antitumoral roles. Thus, further investigation is required to decipher the importance of an aged environment on the outcome of antitumor and immunotherapy responses.

In conclusion, our study found that aging is an important limiting factor for CAR-T cell therapy. Specifically, aged T cells present reduced NAD cellular levels that are linked to decreased mitochondrial fitness, ultimately preventing the maintenance of stem-like properties of CAR-T cells and leading to deficient long-term survival in vivo and tumor growth control. These findings emphasize the importance of using aged models in the field of cancer immunology, which can uncover mechanisms of CAR-T cell failure that are often overlooked in preclinical studies, shedding light on novel strategies that can ameliorate CAR-T cell therapy.

## Methods

### Mice

C57BL/6 CD45.1^+^ and CD45.1 × CD45.2 young (8 weeks old) and old (80–105 weeks old) female mice were bred and maintained in house. For all in vivo experiments, host C57BL/6 CD45.2^+^ female mice (8 weeks old) were purchased from EnVigo laboratories (C57BL/6OlaHsd). Donors and recipients of adoptive T cell transfers were sex-matched. Mice were housed at 22 °C with 55% relative humidity on a 12-h light–dark cycle. Mice were fed ad libitum with Safe-150 chow. All animal experiments were performed in the animal facility in Epalinges at the University of Lausanne (UNIL), as approved by the veterinary authorities of the canton of Vaud and performed in accordance with Swiss federal law (VD3572).

### Cell lines

B16-HER2-mK2 and Phoenix ECO cells were a kind gift from G. Coukos (UNIL) and were cultured in RPMI 1640-Glutamax medium supplemented with 10% heat-inactivated FBS and 1% penicillin–streptomycin. EL4-mCD19 cells were a kind gift from M. L. Davila (Moffitt Cancer Center) and were cultured in RPMI 1640-Glutamax medium supplemented with 10% heat-inactivated FBS and 1% penicillin–streptomycin.

### Preparation of murine CAR-T cells

HER2-directed CAR containing a 41BBz costimulatory domain was cloned in the MSGV retroviral transfer vector as described previously^13,30^. For some experiments, an shRNA for silencing murine *Cd38* was cloned in the HER2_41BBz vector. For retrovirus production, Phoenix ECO cells were transfected with HER2 CAR plasmid and pCL-Eco-packaging plasmid using TurboFect transfection reagent (LifeTechnologies) in OptiMEM medium (Thermo Fisher). After 48 h and 72 h, supernatants were recovered and virus was collected by ultracentrifugation (Beckman Avanti J-26). Spleens from wild-type CD45.1 or CD45.1 × CD45.2 mice were smashed through a 70-μm cell strainer. CD8^+^ T cells were purified using the EasySep mouse CD8^+^ T cell isolation kit (StemCell), according to the manufacturer’s instructions. CD8^+^ T cells were plated at a concentration of 0.5 × 10^6^ cells per ml and activated with Activator CD3/CD28 Dynabeads (Thermo Fisher) at a 2:1 bead-to-cell ratio in the presence of recombinant murine IL-2 (10 IU per ml; PeproTech). T cells were maintained in RPMI 1640-Glutamax medium supplemented with 10% heat-inactivated FBS, 1% penicillin–streptomycin, 5 μM 2-mercaptoethanol (Gibco) and sodium pyruvate (Gibco). T cells were transduced 24 h and 48 h after activation using 48-well plates precoated with RetroNectin (20 μg ml^−1^; Takara). After overnight coating at 4 °C, 48-well plates were blocked for 30 min with 2% BSA in PBS, followed by a PBS wash before adding the concentrated retroviruses. Retroviruses were centrifuged for 90 min at 2,000 rcf and 32 °C. Then, T cells were added on top of the viruses and centrifuged for 10 min at 300 rcf and 25 °C. On day 3, activation beads were removed and T cells were expanded using either mouse IL-2 or human IL-7 and IL-15 (10 ng ml^−1^; Miltenyi Biotec). T cell media and cytokines were replaced on day 5 and transduction efficacy was assessed on day 7. Metabolic and phenotype analyses, as well as ACT for in vivo experiments, were always performed on day 7 unless otherwise stated in the figure legend. Where stated, CAR-T cells were treated with NMN (1 mM; Sigma Aldrich), 78c (200 nM; Sigma Aldrich) and/or olaparib (5 μM; Lubio Science) on days 3 and 5 after activation.

### Flow cytometry

The following conjugated antibodies were used for murine experiments: CD3ε–PercP Cy5.5 (clone 145-2C11, 100328, Biolegend, 1/50) or PB (clone 17A2, Department of Oncology, UNIL, 1/100), CD4–PE Cy5 (clone RM4-5, 15-0042-82, eBioscience, 1/100), CD8β–BUV661 (clone 53.6.7, 376-0081-82, Thermo Fisher, 1/100), CD45.1–PE (clone A20.1, 12-0453-82, Biolegend, 1/1,000), CD45.2–BUV395 (clone 104, 363-0454-82, Thermo Fisher, 1/50), CD44–APC (clone IM.781, 103012, Biolegend, 1/100), CD62L–PECy7 (Mel-14, 25-0621-82, eBio, 1/1,000), Thy1.1–BV605 (clone OX-7, 202537, Biolegend, 1/100), CD38–APCCy7 (clone 90, 102728, Biolegend, 1/200), PD1–BV605 (clone 29F.1A12, 135220, Biolegend, 1/200) or PD1–AF647 (clone 29F.1A12, 135230, Biolegend, 1/200), TIM3–BV421 (clone RMT3-23, 119723, Biolegend, 1/200), LAG3–PercP eFluor710 (clone C9B7W, 46-2231-82, Thermo Fisher, 1/200), TOX–PE (clone REA473, 130-120-716, Miltenyi Biotec, 1/50), IFNγ–APC (clone XMG1.2, 17-7311-82, Thermo Fisher, 1/200) and TNF–FITC (clone MP6-XT22, 506304, Biolegend, 1/200). TCF1 (clone C63D9, 2203S, Cell Signaling, 1/200) was stained with an unconjugated antibody. An additional staining was performed with secondary goat anti-rabbit IgG (4412S, Cell Signaling, 1:250). The following conjugated antibodies were used for human experiments: CD3–BV711 (clone UCHT1, 300464, Biolegend, 1/200), CD4–BV605 (clone OKT4, 317438, Biolegend, 1/200), CD8–APC (clone SK1, 344722, Biolegend, 1/200), CCR7–BV421 (clone G043H7, 353208, Biolegend, 1/100), CD45RA–PE TexasRed (clone MEM-56, MHCD45RA17, Thermo Fisher, 1/50), CD62L–PercP Cy5.5 (clone DREG-56, 304824, Biolegend, 1/200) and CD38–AF700 (clone HIT2, 303524, Biolegend, 1/200).

For assessment of intracellular markers, cells were fixed and permeabilized using FoxP3 fixation and permeabilization buffer (00-5523-00, eBioscience). For live–dead discrimination, live/dead Aqua or live/dead NIR kits were used (L34957 and L10119, LifeTechnologies). To assess mitochondrial activity and mitochondrial size, cells were stained with TMRM (T668; 25 nM) and MitoTracker Green (M7514; 100 nM) for 30 min at 37 °C. For cytokine staining, T cells were restimulated using anti-CD3ε-coated plates for 4 h in the presence of brefeldin A (420601, BioLegend, 1/1,000). For pH2AX staining, cells were stained upon fixation and permeabilization using the PECy7-conjugated anti-H2A.X (S139) antibody for 1 h at 4 °C (613420, Biolegend). The samples were acquired using the CytoFLEX S (Beckman Coulter), CytoFLEX LX (Beckman Coulter) or Aurora (Cytek Biosciences). Data analysis was performed using FlowJo (version 10.9.0).

### In vivo CAR-T experiments

For the HER2_41BBz-B16 model, B16-HER2 (10^5^) cells were subcutaneously injected on the right flank of 8-week-old C57BL/6 CD45.2^+^ mice. After 9 days, mice were exposed to sublethal irradiation (5 Gy) followed by one round of intravenous CAR-T cell transfer (2–3 × 10^6^ cells per mouse) performed on day 10. Before the transfer, mice were randomized to have comparative tumor volumes. For the mCD19_28z-EL4 model, EL4-mCD19 cells (5 × 10^5^) cells were subcutaneously injected into the right flank of 8-week-old C57BL/6 CD45.2^+^ mice. After 6 days, mice were exposed to sublethal irradiation (5 Gy) followed by one round of intravenous CAR-T cell transfer (3 × 10^6^ cells per mouse) performed on day 7. Before the transfer, mice were randomized to have comparative tumor volumes. Mice were monitored three times a week and tumor length (*L*; greatest longitudinal measurement) and width (*W*; greatest transverse measurement) were measured with a caliper. Tumor volumes (*V*) were calculated using the formula: *V* = (*L* × *W*^2^)/2. Mice were killed at endpoint by CO_2_ and, where indicated, tumors, spleens and lymph nodes were collected. As permitted by the Swiss federal law, a maximal tumor size of 1,000 m^3^ was reached. In some cases, this limit was exceeded on the last day of measurement and the mice were immediately killed. No statistical methods were used to predetermine sample sizes but our sample sizes are similar to those reported in previous publications^23,61^. Data collection and analysis were not performed blind to the conditions of the experiment.

### Construction of CD38 OE vector

Murine CD38 complementary DNA was synthesized and flanked with the restriction enzymes Not1 and SalI, which were cloned into a retroviral MSCV vector containing a Thy1.1 promoter. The construction of the CD38 OE vector was performed by GenScript Biotech. The sequence can be found in Supplementary Table 2.

### EM

Young and old naive CD8^+^ T cells were sorted using the EasySep mouse naive CD8^+^ T cell isolation kit (StemCell). For the analysis, EM was performed as described previously^62^. Sorted cells were fixed in their culture medium with glutaraldehyde (EM Sciences) at a final concentration of 2.5% in phosphate buffer (0.1 M PB pH 7.4; Sigma) for 10 min at room temperature (RT). As noted previously, they were directly postfixed by a fresh mixture of glutaraldehyde 2.5%, osmium tetroxide 1% (EM Sciences) and potassium ferrocyanide 1.5% (Sigma) in PB for 1 h at RT. The samples were then washed three times in distilled water and spun down in low-melting-point agarose 2% in H_2_O (Sigma), left to solidify on ice, cut into 1-mm^3^ cubes and dehydrated in acetone solution (Sigma) at graded concentrations (30%, 40 min; 50%, 40 min; 70%, 40 min; 100%, 1 h twice). This was followed by infiltration in Epon (Sigma) at graded concentrations (Epon 1:3 acetone, 2 h; Epon 3:1 acetone, 2 h; Epon 1, 1–4 h; Epon 1, 1–12 h) and finally polymerized for 48 h at 60 °C in oven. Ultrathin sections of 50 nm were cut on a Leica Ultracut (Leica Mikrosysteme) and picked up on a copper slot grid (2 × 1 mm; EM Sciences) coated with a PEI film (Sigma). Sections were poststained with uranyl acetate 2% (Sigma) in H_2_O for 10 min, rinsed several times with H_2_O followed by Reynolds lead citrate in H_2_O (Sigma) for 10 min and rinsed several times with H_2_O. Micrographs were recorded with a transmission EM instrument (Philips CM100, Thermo Fisher Scientific) with a TemCam-F416 digital camera (TVIPS). Image analysis and quantification were carried out using ImageJ software (version 2.16.0). The number of mitochondria per cell was quantified. For assessing mitochondrial cristae, each dot represents the crista number in one mitochondrion from one high-magnitude EM image of a live cell.

### Seahorse XFe96 analysis

OCRs were determined using a Seahorse Bioanalyzer XFe96. Briefly, CD8^+^ T cells were resuspended in Seahorse XF basic medium supplemented with 10 mM glucose, 1 mM sodium pyruvate and 2 mM glutamine (pH 7.4, at 37 °C). CD8^+^ T cells were plated in a Cell-Tak-coated (22.4 μg ml^−1^) Seahorse XFe96 microplate (2 × 10^5^ cells per well). The injection ports were loaded with 1 μM oligomycin, 2 μM carbonyl cyanide-*p*-trifluoromethoxyphenylhydrazone (FCCP) and 0.5 μM rotenone–antimycin. During sensor calibration, cells were incubated in a 37 °C non-CO_2_ incubator for 45 min. Data were analyzed using Seahorse Wave (version 2.4.3).

### Killing assay

To determine the killing capacity of murine CAR-T cells, the IncuCyte ZOOM system was used. Specifically, 10^4^ B16-HER2 cells containing the red fluorescent protein mK2 were plated in flat-bottom 96-well plates. After 4 h of incubation, young or old CAR-T cells expanded with IL-2 or IL-7 and IL-15 were added on top at a 2:1 effector-to-target ratio. Upon coculture, plates were placed immediately in the IncuCyte ZOOM system for 3 days. Killing capacity was determined by quantifying the red area and normalizing to negative controls (that is, B16-HER2 mK2 cells cultured in the absence of CAR-T cells). To evaluate the killing capacity of human CAR-T cells upon multiple rechallenges, 10^3^ NALM6–GFP cells were seeded in a flat-bottom 96-well plate and CAR-T cells were added at an effector-to-target ratio of 1:4. Subsequently, 10^5^ NALM6–GFP cells were added every 2–3 days for repeated tumor challenges. The cytotoxic activity of CAR-T cells was monitored over 23 days by measuring the GFP signal density (objects per mm²) using the IncuCyte ZOOM system.

### Metabolomics

The intracellular level of different metabolites was determined by performing LC–MS/MS analysis. For metabolite extraction, cell lysates were extracted by the addition of methanol and H_2_O (4:1), followed by homogenization with ceramic beads in the Cryolys Precellys 24 sample homogenizer. Homogenized extracts were then centrifuged for 15 min at 4,000*g* and 4 °C and the resulting supernatant was collected and evaporated to dryness in a vacuum concentrator. Dried extracts were resuspended in methanol and H_2_O before LC–MS/MS analysis. Raw LC–MS/MS data were processed using the Agilent Quantitative analysis software. Raw data can be found in Supplementary Table 1.

### NAD ELISA

For NAD and reduced NAD (NADH) quantification, the NAD/NADH quantitation kit (SigmaAldrich) was used. For each sample, 1 × 10^6^ cells per sample were collected and the manufacturer’s instructions were followed.

### mtDNA and nuclear DNA (nDNA) measurement

DNA was isolated using the DNeasy kit (Qiagen), according to the manufacturer’s instructions. To determine the mtDNA-tonDNA ratio, qPCR was performed using Power SYBR green master mix (Thermo Fisher) and analyzed on the 7900HT system (Applied Biosystems) to estimate the relative values for mtDNA (*COX1*) and nDNA (*NDUFV1*).

Forward *COX1*: 5′-TGCTAGCCGCAGGCATTAC-3′

Reverse *COX1*: 5′-GGGTGCCCAAAGAATCAGAAC-3′

Forward *NDUFV1*: 5′-CTTCCCCACTGGCCTCAAG-3′

Reverse *NDUFV1*: 5′-CCAAAACCCAGTGATCCAGC-3′

### In vitro exhaustion model

CD8^+^ T cells were isolated and activated using plates coated with Ultra-LEAF anti-mouse CD3e (3 μg ml^−1^, Biolegend) and soluble Ultra-LEAF anti-mouse CD28 (1.5 μg ml^−1^, Biolegend). After 3 days, cells were washed and expanded for 4 days in the presence of IL-7 and IL-15. On days 7, 9 and 11, CD8 T cells were restimulated using CD3-coated plates. Fresh medium and IL-7 and IL-15 were added to the wells at the time of restimulation. Where indicated, cells were treated with NR (1 mM) on days 7, 9 and 11. Phenotypic and functional readouts were performed on day 12.

### Preparation of human CAR-T cells

Human blood samples were obtained from healthy young donors (aged 25–28 years) and elderly donors (aged 66–70 years). To generate CAR-T cells, CD3^+^ T cells were negatively isolated using the RosetteSep human T cell enrichment cocktail (StemCell, 15061). T cells were treated with ACK lysis buffer (Gibco, A10492-01) and resuspended in RPMI 1640 medium (Gibco, 21875158) supplemented with 10% FBS, 1% penicillin–streptomycin, 1 mM sodium pyruvate (Gibco, 11360-070) and 10 mM HEPES (Gibco, 15630-056). For stimulation, T cells were cultured with CD3/CD28-activating Dynabeads (Gibco, 11132D) at a 1:1 bead-to-cell ratio. The following day, T cells were transduced with the hCD19_41BBz (FMC63 scFv) or hCD19_CD28z lentiviral vector at a multiplicity of infection of 4. Recombinant human IL-2 (Proleukin, Roche) or a cytokine combination of IL-2, IL-7 (Peprotech, 200-07) and IL-15 (Peprotech, 200-15) was added every other day to a final concentration of 30 IU per ml and 10 ng ml^−1^, respectively. Cells were counted and fed every 2 days until day 10, after which they were cryopreserved. All T cell functional assays were conducted in media without cytokines. For the generation of CAR-T cells from participants with melanoma, 2 × 10^5^ frozen PBMCs from young (aged 40–44 years) and elderly (aged 79–84 years) donors were seeded in a round-bottom 96-well plate and processed as described above.

Donations from healthy volunteers were approved by the Comission Cantonale d’Ethique de la Recherche Genève (CCER). Samples from participants with cancer were taken from a biobank supported by the study protocol CCER 2016-01237. Written informed consent was obtained from all individuals.

### Reanalysis of public single-cell RNA sequencing (scRNA-seq) data

An scRNA-seq dataset of human baseline PBMCs from 20 participants with LBCL was obtained from the Gene Expression Omnibus (GSE197268). Metadata on age and response to therapy were obtained from the supplementary material of the associated publication^49^. To exclude low-quality cells and outliers, the following quality control filters were applied on the scRNA-seq data: percentage of mitochondrial genes < 25%, number of detected genes = 300–5,000, number of UMIs = 500–20,000 and log_10_ genes per UMI > 0.6. Raw counts were normalized using a standard log1p normalization implemented in Seurat (version 5.0.1)^63^. Broad cell types for each scRNA-seq sample were predicted using the scGate tool and its default PBMC model^64^. CD8 T cell subtypes were annotated using ProjecTILs^65^ and a published reference map of human CD8 T cell subtypes^66^. Gene signatures for NAD metabolism and DNA repair were downloaded from MSigDB^67^ under the ‘Gene Ontology biological process’ subset. On the basis of these gene sets, signature scores were calculated using the UCell method with default parameters^68^. Statistical comparisons (Wilcoxon test) were performed at the sample level by averaging signature scores for each participant and cell type. To assess the expression of CD38 in the context of markers of stemness and exhaustion, we interrogated two multistudy reference maps of murine^65^ and human^66^ tumor-infiltrating T cells. For each T cell subtype, we calculated the normalized average expression of *CD38*, *HAVCR2*, *TOX* and *TCF7*.

### Statistical analysis

All statistical analyses were performed using GraphPad Prism version 10.0.3. The sample size (*n*) is stated in each figure legend together with the statistical test adopted. Data are shown as the mean ± s.e.m. *P* values < 0.05 were considered significant. Data distribution was assumed to be normal but this was not formally tested. All statistical tests used were two-sided.

### Reporting summary

Further information on research design is available in the Nature Portfolio Reporting Summary linked to this article.

## Supplementary information

Reporting Summary

Supplementary Table 1 Supplementary Table 1: Metabolomic raw data. Supplementary Table 2: Sequence for CD38 OE vector.

## Source data

Source Data Fig. 1 Statistical source data.

Source Data Fig. 2 Statistical source data.

Source Data Fig. 3 Statistical source data.

Source Data Fig. 4 Statistical source data.

Source Data Fig. 5 Statistical source data.

Source Data Extended Data Fig. 1 Statistical source data.

Source Data Extended Data Fig. 3 Statistical source data.

Source Data Extended Data Fig. 4 Statistical source data.

Source Data Extended Data Fig. 5 Statistical source data.

Source Data Extended Data Fig. 6 Statistical source data.

Source Data Extended Data Fig. 7 Statistical source data.

Source Data Extended Data Fig. 8 Statistical source data.

## Data Availability

scRNA-seq data from the previous study^49^ are publicly available from the Gene Expression Omnibus under accession number GSE197268. RNA-seq data from the previous study^50^ are publicly available online (https://tanlab4generegulation.shinyapps.io/Tcell_Atlas/). The remaining data are available within the article and Supplementary Information or from the corresponding authors upon request. Source data are provided with this paper.
