# Supplementary material for: Age-associated nicotinamide adenine dinucleotide decline drives CAR-T cell failure
Source: Nat Cancer. 2025 May 20;6(9):1524–36. doi: 10.1038/s43018-025-00982-7 (PMC12463664; doi:10.1038/s43018-025-00982-7)
Supplement: Supplementary file 1 — Reporting Summary [file 43018_2025_982_MOESM1_ESM.pdf]

nature portfolio

Corresponding author(s): Nicola Vannini
Helen Carrasco Hope
Denis Migliorini

Last updated by author(s): 4/15/2025

Reporting Summary

Nature Portfolio wishes to improve the reproducibility of the work that we publish. This form provides structure for consistency and transparency in reporting. For further information on Nature Portfolio policies, see our Editorial Policies and the Editorial Policy Checklist.

Statistics

For all statistical analyses, confirm that the following items are present in the figure legend, table legend, main text, or Methods section.

n/a	Confirmed	
☐	☒	The exact sample size (*n*) for each experimental group/condition, given as a discrete number and unit of measurement
☐	☒	A statement on whether measurements were taken from distinct samples or whether the same sample was measured repeatedly
☐	☒	The statistical test(s) used AND whether they are one- or two-sided<br>*Only common tests should be described solely by name; describe more complex techniques in the Methods section.*
☒	☐	A description of all covariates tested
☐	☒	A description of any assumptions or corrections, such as tests of normality and adjustment for multiple comparisons
☐	☒	A full description of the statistical parameters including central tendency (e.g. means) or other basic estimates (e.g. regression coefficient) AND variation (e.g. standard deviation) or associated estimates of uncertainty (e.g. confidence intervals)
☐	☒	For null hypothesis testing, the test statistic (e.g. *F*, *t*, *r*) with confidence intervals, effect sizes, degrees of freedom and *P* value noted<br>*Give P values as exact values whenever suitable.*
☒	☐	For Bayesian analysis, information on the choice of priors and Markov chain Monte Carlo settings
☒	☐	For hierarchical and complex designs, identification of the appropriate level for tests and full reporting of outcomes
☒	☐	Estimates of effect sizes (e.g. Cohen's *d*, Pearson's *r*), indicating how they were calculated

Our web collection on statistics for biologists contains articles on many of the points above.

Software and code

Policy information about availability of computer code

Data collection	SpectroFlo, CytExpert, 7500 Fast real time PCR system, Incucyte
Data analysis	FlowJo 10.9.0, GraphPad Prism 10.0.3, SeaHorse Wave 2.4.3, Microsoft Excel 16.77.1, ImageJ 2.16.0

For manuscripts utilizing custom algorithms or software that are central to the research but not yet described in published literature, software must be made available to editors and reviewers. We strongly encourage code deposition in a community repository (e.g. GitHub). See the Nature Portfolio guidelines for submitting code & software for further information.

Data

Policy information about availability of data

All manuscripts must include a data availability statement. This statement should provide the following information, where applicable:

- Accession codes, unique identifiers, or web links for publicly available datasets
- A description of any restrictions on data availability
- For clinical datasets or third party data, please ensure that the statement adheres to our policy

Single-cell RNA-seq data from the previous study (Haradhvalal et al. 2022, Nat Med) are publicly available at Gene Expression Omnibus with GEO accession GSE197268 [https://www.ncbi.nlm.nih.gov/geo/query/acc.cgi?acc=GSE197268]. RNA-seq data from the previous study (Chen et al. 2021, Cancer Discov) are publicly available at the online platform https://tanlab4generegulation.shinyapps.io/Tcell_Atlas/. The remaining data are available within the Article, Supplementary Information, Source Data file and/or from the corresponding authors upon request. Source data are provided with this paper.

Human research participants

Policy information about studies involving human research participants and Sex and Gender in Research.

Reporting on sex and gender	Sex was considered in the study designed, and sex-matched comparisons were performed. All participants gave consent.
Population characteristics	For some experiments, human PBMCs were extracted from healthy donors. Young donors ranged 25-29 years old. Old donors ranged 66-70 years old.<br>For some experiments, human PBMCs from non-small cell lung cancer (NSCLC) and melanoma patients were used. Patients were aged between 39 to 85 years old.
Recruitment	Donations from healthy volunteers were approved by the Comission Cantonale d'ethique de la recherche Genève (CCER). Samples from patients with cancer were taken from a biobank supported by the study protocol CCER 2016-01237. Written informed consent was obtained from all individuals.
Ethics oversight	Commission Cantonale d'éthique de la recherche Genève (CCER).

Note that full information on the approval of the study protocol must also be provided in the manuscript.

Field-specific reporting

Please select the one below that is the best fit for your research. If you are not sure, read the appropriate sections before making your selection.

☒ Life sciences ☐ Behavioural & social sciences ☐ Ecological, evolutionary & environmental sciences

For a reference copy of the document with all sections, see nature.com/documents/nr-reporting-summary-flat.pdf

Life sciences study design

All studies must disclose on these points even when the disclosure is negative.

Sample size	No statistical method was used to determine sample size. Sample sizes were determined based on our previous experience and pilot experiments thus taking in consideration the inter sample variability.
Data exclusions	Some mice were excluded due to splenomegaly.
Replication	Data were successfully reproduced at least 2 or 3 times.
Randomization	For in vivo experiments, mice were allocated randomly to each experimental group, ensuring equal tumor volume in all groups before treatment. For in vitro experiments (both mice and human), T cells derived from the same donor were splitted into several conditions and compared.
Blinding	For tumor measurements, performed with a caliper, and quantification of mitochondrial morphology by EM, investigators were blind.

Reporting for specific materials, systems and methods

We require information from authors about some types of materials, experimental systems and methods used in many studies. Here, indicate whether each material, system or method listed is relevant to your study. If you are not sure if a list item applies to your research, read the appropriate section before selecting a response.

Materials & experimental systems

n/a	Involved in the study	
☐	☒	Antibodies
☐	☒	Eukaryotic cell lines
☒	☐	Palaeontology and archaeology
☐	☒	Animals and other organisms
☒	☐	Clinical data
☒	☐	Dual use research of concern

Methods

n/a	Involved in the study	
☒	☐	ChIP-seq
☐	☒	Flow cytometry
☒	☐	MRI-based neuroimaging

Antibodies

Antibodies used	The following conjugated antibodies were used for murine experiments: CD3⍰ - PercP Cy5.5 (clone 145-2C11, #100328, Biolegend, 1/50) or PB (clone 17A2, Department of Oncology, UNIL, 1/100), CD4 – PE Cy5 (clone RM4-5, #15-0042-82, eBioscience, 1/100) ,CD8⍰ - BUV661 (clone 53.6.7, #376-0081-82, Thermo Fisher, 1/100), CD45.1 – PE (clone A20.1, #12-0453-82, Biolegend, 1/1000), CD45.2 – BUV395 (clone 104, #363-0454-82, Thermo Fisher, 1/50), CD44 – APC (clone IM.781, #103012, Biolegend, 1/100), CD62L – PECy7 (Mel-14, #25-0621-82, eBio, 1/1000), Thy1.1 – BV605 (clone OX-7, #202537, Biolegend, 1/100), CD38 – APCCy7 (clone 90, #102728, Biolegend, 1/200), PD1 – BV605 (clone 29F.1A12, #135220, Biolegend, 1/200) or AF647 (clone 29F.1A12, #135230, Biolegend, 1/200), TIM3 – BV421 (clone RMT3-23, #119723, Biolegend, 1/200), LAG3 – PercP eFluor710 (clone C9B7W, #46-2231-82, Thermo Fisher, 1/200), TOX – PE (clone REA473, #130-120-716, Miltenyi Biotec, 1/50), IFN⍰ - APC (clone XMG1.2, #17-7311-82, Thermo Fisher, 1/200), TNF⍰ - FITC (clone MP6-XT22, #506304, Biolegend, 1/200), TCF-1 (clone C63D9, #2203S, Cell Signaling, 1/200) was stained with an unconjugated antibody. An additional staining was performed with secondary goat Anti-Rabbit IgG (#4412S, Cell Signaling, 1:250). The following conjugated antibodies were used for human experiments: CD3 – BV711 (clone UCHT1, #300464, Biolegend, 1/200), CD4 – BV605 (clone OKT4, #317438, Biolegend, 1/200), CD8 – APC (clone SK1, #344722, Biolegend, 1/200), CCR7 – BV421 (clone G043H7, #353208, Biolegend, 1/100), CD45RA - PE TexasRed (clone MEM-56, #MHCD45RA17, Thermo Fisher, 1/50), CD62L – PercP Cy5.5 (clone DREG-56, #304824, Biolegend, 1/200), CD38 – AF700 (clone HIT2, #303524, Biolegend, 1/200).). For pH2AX staining, cells were stained upon fix/perm using the PECy7 conjugated antiH2A.X (Ser139) antibody for 1h at 4⍰C (#613420, Biolegend).
Validation	All the antibodies have been tested and validated previously by our Flow Cytometry Facility at Ludwig Cancer Institute of Lausanne.

Eukaryotic cell lines

Policy information about cell lines and Sex and Gender in Research

Cell line source(s)	PhoenixECO and B16-HER2-mK2 cells were a gift from the laboratory of Prof. George Coukos (UNIL). EL4-mCD19 cells were a gift from Marco L. Davila laboratory (Moffitt Cancer Centre, Florida).
Authentication	None of the cell lines used were authenticated, but low passage number cell lines were used.
Mycoplasma contamination	Cell lines were not tested for Mycoplasma.
Commonly misidentified lines (See ICLAC register)	N/A

Animals and other research organisms

Policy information about studies involving animals; ARRIVE guidelines recommended for reporting animal research, and Sex and Gender in Research

Laboratory animals	We used C57Bl/6 mice, exploiting the double congenic allelic system (CD45.1, CD45.2 and CD45.1/2). Young mice were 8-weeks old, while old mice were always >80 weeks old. For in vivo experiments, host mice were purchased from EnVigo laboratories (C57Bl/6OlaHsd). All mice were housed in conventional animal facility of University of Lausanne and were kept in ventilated cages, at 22 C with 55% humidity and a 12 hour dark/light cycle.
Wild animals	None
Reporting on sex	Sex was not considered in the study
Field-collected samples	No field-collected samples were used
Ethics oversight	All animal experiments were performed in the animal facility in Epalinges at the University of Lausanne (UNIL), as approved by the veterinary authorities of the canton of Vaud and performed in accordance with Swiss Federal Law (VD3572).

Note that full information on the approval of the study protocol must also be provided in the manuscript.

Flow Cytometry

Plots

Confirm that:

☒ The axis labels state the marker and fluorochrome used (e.g. CD4-FITC).

☒ The axis scales are clearly visible. Include numbers along axes only for bottom left plot of group (a 'group' is an analysis of identical markers).

☒ All plots are contour plots with outliers or pseudocolor plots.

☒ A numerical value for number of cells or percentage (with statistics) is provided.

Methodology

Sample preparation

Cell suspensions from the spleen were obtained by mashing through a 40µM nylon cell strainer, followed by red blood cells lysis using ACK buffer. Surface staining was performed with mAbs for 20 min at 4°C in PBS supplemented with 2% FCS (FACS buffer).
For intranuclear staining, cells were surface stained before fixation and permeabilization using the Foxp3 transcription factor staining kit (eBioscience: Cat. No. 00-5523) followed by intranuclear staining in Permeabilization buffer 1x (Perm buffer).
For the detection of cytokine production, CD8+ T cells were re-stimulated in vitro with anti-CD3 (3ug/ml) in the presence of Brefeldin A (5µg/ml) for the last 4h.

Instrument

CytoFLEX S (Beckman Coulter), CytoFLEX LX (Beckman Coulter) or Aurora (Cytek Biosciences)

Software

CytExpert, SpectroFlo. FlowJo v10.9.0 was used for data analysis.

Cell population abundance

For in vitro experiments, CAR-T cells were generated from isolated CD8+ T cells from spleen, with a purity > 90%. Characterization of CAR-T cells was performed upon expansion within viable CD8+ T cells, which represented the majority f the analyzed cells. For in vivo experiments, CAR-T cells were detected in spleens upon processing. Cell population represented 0.1-0.5% of total splenocytes, depending on the sample.

Gating strategy

Gating was based on single staining control and published literature

☒ Tick this box to confirm that a figure exemplifying the gating strategy is provided in the Supplementary Information.
